# Advances in moyamoya disease: pathogenesis, diagnosis, and therapeutic interventions

**DOI:** 10.1002/mco2.70054

**Published:** 2025-01-14

**Authors:** Shihao He, Zhenyu Zhou, Michelle Y. Cheng, Xiaokuan Hao, Terrance Chiang, Yanru Wang, Junze Zhang, Xilong Wang, Xun Ye, Rong Wang, Gary K. Steinberg, Yuanli Zhao

**Affiliations:** ^1^ Department of Neurosurgery Peking Union Medical College Hospital, Peking Union Medical College and Chinese Academy of Medical Sciences Beijing China; ^2^ Department of Neurosurgery Stanford University School of Medicine Stanford California USA; ^3^ Department of Neurosurgery Beijing Tiantan Hospital, Capital Medical University Beijing China; ^4^ Department of Pathology Stanford University School of Medicine Stanford California USA

**Keywords:** genetic susceptibility, immunity, moyamoya disease, pathogenesis, pathological angiogenesis

## Abstract

Moyamoya disease (MMD) is a type of cerebrovascular disease characterized by occlusion of the distal end of the internal carotid artery and the formation of collateral blood vessels. Over the past 20 years, the landscape of research on MMD has significantly transformed. In this review, we provide insights into the pathogenesis, diagnosis, and therapeutic interventions in MMD. The development of high‐throughput sequencing technology has expanded our understanding of genetic susceptibility, identifying MMD‐related genes beyond RNF213, such as ACTA2, DIAPH1, HLA, and others. The genetic susceptibility of MMD to its pathological mechanism was summarized and discussed. Based on the second‐hit theory, the influences of inflammation, immunity, and environmental factors on MMD were also appropriately summarized. Despite these advancements, revascularization surgery remains the primary treatment for MMD largely because of the lack of effective in vivo and in vitro models. In this study, 16 imaging diagnostic methods for MMD were summarized. Regarding therapeutic intervention, the influences of drugs, endovascular procedures, and revascularization surgeries on patients with MMD were discussed. Future research on the central MMD vascular abnormalities and peripheral circulating factors will provide a more comprehensive understanding of the pathogenic mechanisms of MMD.

## Graphical abstract

The general morphology of moyamoya disease is described in the illustrated summary. The hyperplasia of the intima was found in the pathological tissue. In addition, the main research results of this review from genes, proteomics, metabolomics, immune involvement, and pathogenesis are summarized.

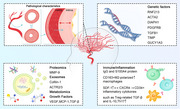

## 1 INTRODUCTION

Moyamoya disease (MMD) is a rare occlusive cerebrovascular disease. First described by Suzuki and Takaku in Japan in 1969, the disease was named “moyamoya” owing to the appearance of smoke‐like cerebral vessels in cerebrovascular images. MMD is characterized by occlusion of the terminal end of the internal carotid artery and compensatory proliferation of intracranial vessels.^1^ Although the mechanisms underlying the pathogenesis of MMD remain unclear, ongoing research continues to uncover the intricate biological processes involved, advancing our understanding of this complex disease.

MMD is more frequently observed in East Asia. Therefore, most of the existing epidemiological studies on MMD have been carried out in this region. The onset of MMD has two age peaks: at 10 years and 40 years of age.^2^ There is a marked disparity in the age distribution between males and females: for males, it is 10–14 years old, and for females, it is 20–24 years old.^3^ In Japan, the incidence is 0.35–0.94 per 100,000 person‐years, and the prevalence is 3.16 per 100,000 individuals.^4^ In two large‐scale studies on MMD in Japan, the male‐to‐female ratio was approximately 1.9:1, among which 11%–12% had a family history of MMD.^2^, ^3^ In China, in a study that included 4128 patients with MMD, the ratio of male‐to‐female patients was 1:1. In northern and northeastern China, ischemic MMD is predominant, and transient ischemic attacks (TIAs) involve the longest initial symptoms.^5^ In the United States, the racial distribution of patients with MMD is 49% Caucasian, 24% African American, 11% Asian, and 11% Hispanic.^6^, ^7^ However, the incidence and prevalence of the disease are highest among Asians, especially among Japanese.

Since the discovery of MMD, research methodologies have continuously evolved and improved. Over the past two decades, the advent of high‐throughput sequencing has propelled biological research into a new era. Knowledge on the pathology, diagnosis, and treatment of MMD often substantially change within a few years.^8^ Initially, RNF213 was identified as the primary susceptibility gene for MMD; however, other genes have since been linked to this disease.^9^

While a successful animal model for MMD has yet to be developed, recent progress has been made with cellular models that replicate the key pathological characteristics of MMD.^10^, ^11^ The differential diagnose of MMD and moyamoya syndrome (MMS) are also constantly evolving.^8^, ^9^, ^12^ In the 1970s, the Research Committee on Moyamoya Disease (RCMD) of Japan offered approaches for the diagnosis, treatment, and prevention of MMD. In 1978, the first guidelines for the diagnosis and treatment of MMD were formulated, and up to now, there have been five editions.^13^ The latest RCMD guidelines have eliminated the diagnostic condition that the bilateral intracranial carotid arteries must be involved in the previous definitions.^12^, ^13^ Currently, the involvement of the proximal middle cerebral artery or the anterior cerebral artery is sufficient for the diagnosis of MMD. Observation studies have revealed that there is a growing number of patients with MMD progressing from unilateral to bilateral, thus unilateral cases can also be diagnosed as MMD.

To date, no studies have reported that drugs can reverse the pathological progression of MMD. Current pharmacological treatments mainly address the clinical symptoms of MMD, such as cerebral infarction and cerebral hemorrhage.

Existing studies have proved that antiplatelet therapy has long‐term benefits for patients with MMD. The treatment guidelines in Japan in 2012 have incorporated antiplatelet drugs into the treatment plan for ischemic MMD.^14^ Endovascular treatment has not been recommended for therapeutic intervention of MMD as of yet. Currently, it is commonly acknowledged that revascularization surgery is an effective therapeutic intervention for MMD. Revascularization surgery is generally classified as: direct, indirect, and combined approaches. It is essential to pay attention to the temporary neurological dysfunction caused by changes in cerebral hemodynamics after the operation.^15^ Some studies have indicated that approximately 25% of patients undergoing direct surgical procedures for MMD will present with symptoms of hyperperfusion.^16^ At present, it is generally considered that elevated preoperative cerebral blood volume (CBV) and oxygen extraction fraction (OEF) are high‐risk factors for postoperative hyperperfusion symptoms.^17^ At present, revascularization surgery has a definite preventive effect on patients with MMD. Therefore, it is currently widely accepted that both ischemic and hemorrhagic MMD patients should receive surgical treatment.^18^

To illustrate the development of research on MMD over the past two decades, we discuss it below in terms of pathogenesis, diagnosis, and therapeutic interventions. Owing to its unclear pathogenesis, the diagnosis of and therapeutic interventions for MMD have always been controversial. A successful experimental model can greatly promote exploration of the pathogenesis of this disease. Regarding the current progress in MMD experimental models, organoids may be the most promising research hotspot for the future.

## 2 PATHOLOGICAL CHARACTERISTICS OF MOYAMOYA DISEASE

**FIGURE 1:**
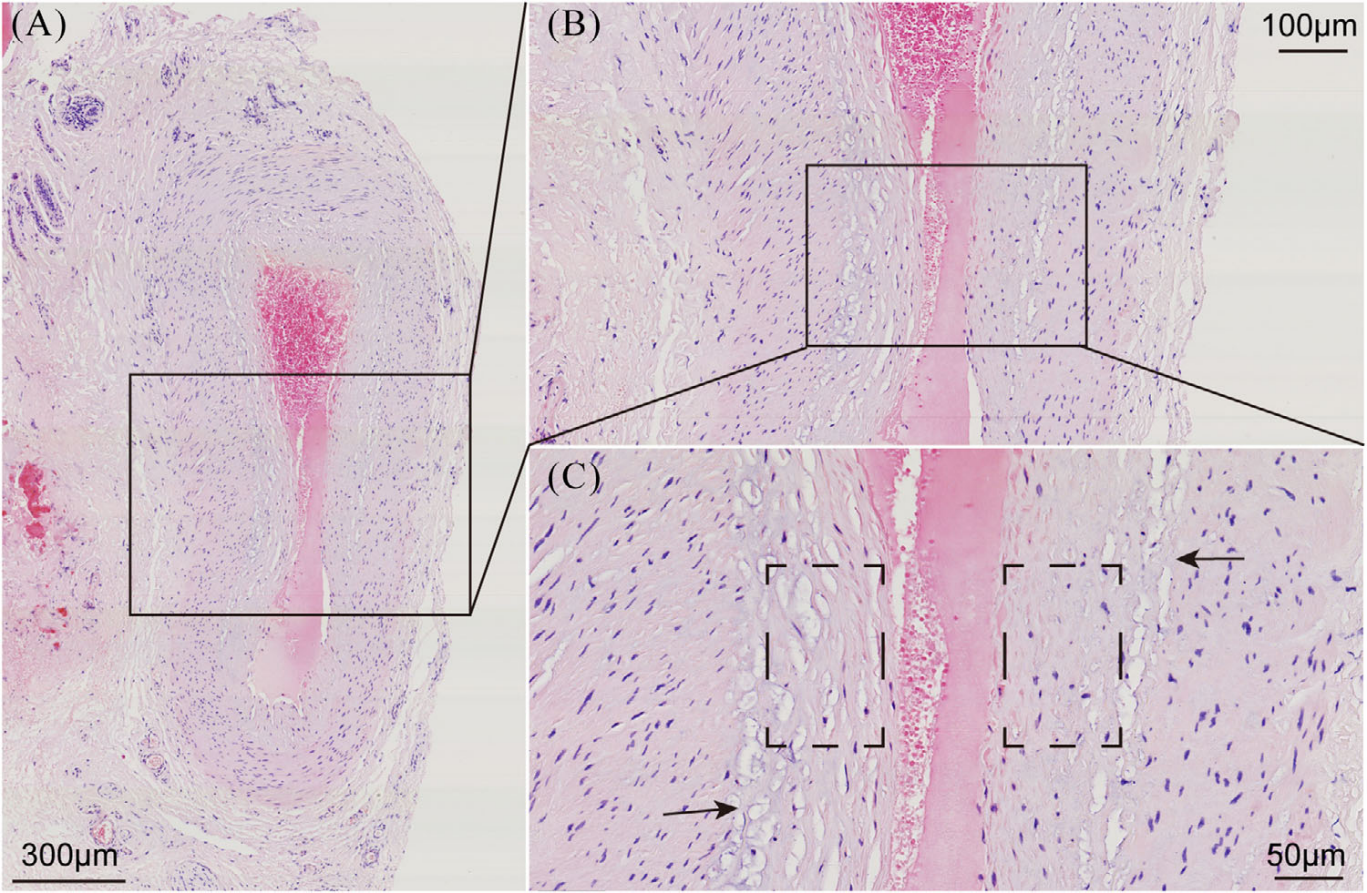
Superficial temporal artery intimal thickening causing hemadostenosis. (A) In moyamoya disease, the vessel lumen is markedly narrowed. Scale bar: 300 µm. (B) The wall of the stenotic vessel is notably thickened. Scale bar: 100 µm. (C) The intima is hyperplastic, and the cells are arranged in a disordered fashion. The structure of the internal elastic membrane is changed. Scale bar: 50 µm.

MMD is characterized by the spontaneous progressive bilateral occlusion of the terminal internal carotid artery and its proximal branches, with compensatory development of collateral vasculature.^1^ Pathological examinations of the affected intracranial vessels in patients with MMD have revealed evidence of fibrocellular intimal thickening with an increased number of smooth muscle cells (SMCs), irregular undulation (waving) of the internal elastic lamina, and attenuation of the media without arteriosclerotic or inflammatory changes^19^, ^20^, ^21^, ^22^ (Figure 1). A previous study has shown that caspase‐3‐dependent apoptosis in middle cerebral arteries may be associated with these histopathological changes.^23^ Moyamoya collaterals are dilated, perforating arteries consisting of a combination of preexisting and newly developed vessels.^1^, ^24^ Pathological analysis of these collaterals has revealed fragmented elastic lamina, thinned media, microaneurysm formation, collapsed vessels, and related thrombosis.^24^, ^25^, ^26^ In 1991, Bo et al. conducted a pathological observational study of the superficial temporal artery (STA) in 15 patients with MMD. Under light microscopy, they found that the inner membrane of the STA was thickened and the elastic layer was broken or even absent. Electron microscopy revealed proliferating SMCs in the inner membrane of the STA, with a large amount of deposited basement membrane–like substance.^27^ These histopathological alterations could potentially be closely associated with the emergence of ischemic and hemorrhagic stroke in patients with MMD.

### 2.1 Proteomic insights into moyamoya disease: Serum and cerebrospinal fluid studies

Research on MMD proteomics has mostly focused on the serum and cerebrospinal fluid (CSF) of patients with MMD. A recent study has revealed a significant decrease in matrix metalloproteinase (MMP)‐9 release in the plasma of these patients. Because MMP‐9 plays a major role in extracellular matrix (ECM) degradation and in the destruction of the basement membrane, causing endothelial cell (EC) migration during angiogenesis, the authors speculated that the decrease in MMP‐9 may be associated with an increase in ECM in MMD lesions, suggesting a disrupted mechanism of angiogenesis.^28^ Lu et al. have reported that the level of MMP‐9 in the serum of patients with hemorrhagic MMD is higher than that in patients with ischemic MMD, indicating that MMP‐9 may serve as a biomarker for predicting hemorrhage in MMD.^29^ In our previous study, we used data‐independent acquisition proteomic analysis to identify the differentially expressed proteins commonly present in different MMD subgroups, including 14,726 peptides and 1555 proteins. This study provided a new interpretation of intimal hyperplasia in patients with MMD by validating the roles of FLNA and ZYX in MMD cell models and demonstrating the importance of protein regulation in the development of MMD.^30^ Using data‐independent acquisition proteomic analysis, Wang et al. have reported a decrease in apolipoprotein C‐I, apolipoprotein D, and apolipoprotein A‐IV levels in the serum of patients with MMD. Their proteomics and enzyme‐linked immunosorbent assay (ELISA) results suggested that the three apolipoproteins are vital factors in the decrease in high‐density lipoprotein level in MMD and that the downregulation of lipid antioxidant function (represented by high‐density lipoprotein) is potentially related to vascular changes in MMD.^31^ Proteomic analysis of the CSF of patients with MMD using two‐dimensional (2D) gel electrophoresis followed by mass spectrometry has revealed an increase in cellular retinoic acid‐binding protein (CRABP)‐I.^32^ Maruwaka et al. investigated the CSF of patients with MMD using surface‐enhanced laser desorption/ionization time‐of‐flight mass spectrometry, and the results indicated that a peptide of 4473 Da might be a reliable biomarker for MMD. The peptide also peaked in younger patients with MMD, and it was closely associated with postoperative angiogenesis.^33^

Recently, exosomes have become a new direction for research on potential biomarkers and the pathogenesis of MMD. He et al. used the GeneChip WT Pico Kit to conduct a comprehensive transcriptome analysis of the peripheral blood of patients with MMD. The results revealed the RNA profile of exosomes of these patients and identified 1486 downregulated and 2405 upregulated differentially expressed RNAs. Additionally, the authors reported that the overexpression of IPO11 and PRMT1 circRNAs could potentially be associated with MMD angiogenesis, whereas the decreased expression of CACNA1F circRNAs may be linked to vascular occlusion. To our knowledge, this was the first study to show that differentially expressed exosomal RNAs are involved in the pathogenesis of MMD.^34^

Wang et al. detected serum‐derived exosomes in pure ischemic or hemorrhagic MMD cases using tandem mass tag‐based quantitative proteomics. They described disrupted actin dynamics in MMD, with cofilin‐1 and actin‐related protein 2/3 downregulated in the exosomes of patients with MMD. After treatment with these exosomes, vascular ECs from the mouse brain showed a higher level of proliferation and more ethynyl‐2‐deoxyuridine‐positive cells than did those in the control group. The authors also suggested that exosomes derived from individuals with hemorrhagic MMD may impair mitochondrial function in mouse cerebrovascular ECs.^35^

In summary, proteins are biological effectors that play important roles in the pathogenesis of MMD. Proteomic analyses have been successfully used to map protein profiles in both the blood and CSF of patients with MMD, uncovering previously unknown functions. These findings not only advance our understanding of MMD but also open new avenues for exploring the pathogenesis of related diseases.

### 2.2 Metabolomic study of biomarkers and pathogenic pathways in moyamoya disease

A recent study used hydrogen‐1 nuclear magnetic resonance spectroscopy to investigate the metabolites associated with MMD in the CSF. Elevated levels of glutamine and taurine were observed in the CSF of patients with MMD compared to that of patients with atherosclerotic stenosis, potentially linking these findings to the pathogenesis of MMD in both bilateral and unilateral adult cases.^36^ Untargeted gas chromatography mass spectrometry revealed 15 upregulated and nine downregulated metabolic biomarkers in the serum of patients with MMD. These biomarkers were involved in several pathways and associated with the metabolism of amino acids, lipids, and carbohydrates.^37^ Utilizing ultra‐high‐performance liquid chromatography coupled with high‐resolution mass spectrometry for untargeted metabolomic analysis, He et al. identified 887 and 510 unique metabolites in ischemic MMD, hemorrhagic MMD, atherosclerotic stenosis, and healthy controls. To date, this study presents the most comprehensive metabolic data on MMD. Furthermore, Lysophosphatidylcholine (LPC) 16:1 is downregulated in patients with ischemic MMD, suggesting that LPC 16:1 may be a candidate biomarker for identifying different subtypes of MMD. LPC supplementation can inhibit the angiogenic function of HBVSMCs, providing a new approach to treating MMD.^38^ Michele et al. conducted an untargeted lipidomic analysis that demonstrated a reduction in membrane complex glycosphingolipids in the plasma of patients with MMD, indicating potential cerebral cellular recruitment. Additionally, using a quantitative targeted lipidomic approach, they observed an elevation in free sphingoid bases, which may be associated with dysregulated angiogenesis.^28^ Targeted metabolomic analysis of amino acid profiles in the serum of patients with MMD also suggested that four amino acids (L‐methionine, L‐glutamic acid, β‐alanine, and o‐phosphoserine) could act as potential biomarkers for the early diagnosis or treatment of MMD.^39^

### 2.3 Factors in pathologic angiogenesis in moyamoya disease

Vascular occlusion and proliferation are the most important pathological changes associated with MMD. Imbalanced angiogenesis, which is also a pathological feature of MMD, is an important cause of pathological changes in the vasculature. Understanding the factors contributing to pathological angiogenesis is pivotal in addressing the challenges within the field of MMD.

#### 2.3.1 RNF213 R4810K

Mysterin (RNF213) R4810K (rs112735431: G > A; referred to in this article as RNF213 R4810K) was identified as a mutation predisposing East Asian individuals to MMD.^40^ The association between RNF213 R4810K and angiogenesis has been extensively explored in numerous studies. Toshiaki et al. developed an induced pluripotent stem cell (iPSC) model using samples from three patients with MMD and three healthy controls and revealed that iPSCs obtained from individuals who are heterozygous or homozygous for RNF213 R4810K exhibit markedly reduced angiogenic activity. The authors overexpressed RNF213 R4810K in human umbilical vein ECs (HUVECs) via plasmid transfection and observed reduced angiogenic activity and proliferation. Moreover, RNF213 R4810K overexpression also significantly reduced the expression of securin (a key player in mitosis). In a subsequent experiment involving securin depletion, the angiogenic activity of the HUVECs was reduced.^41^ They finally demonstrated that the overexpression of RNF213 R4810K delayed mitosis by adversely affecting the localization of mitotic arrest deficient 2 to the kinetochore.^42^ Thus, RNF213 R4810K may inhibit angiogenesis by inducing mitotic abnormalities and increasing the risk of genomic instability. Hatasu et al. found that RNF213 can inhibit angiogenesis by stabilizing the oligomeric states of RNF213 via the inhibition of ATP hydrolysis, which disrupts ATPase activity. They demonstrated that NF213 R4810K (but not wild‐type RNF213) decreased tube formation and the ability of ECs to migrate. Therefore, the authors postulated that RNF213 trapped in the oligomeric state may lead to low angiogenic activity.^43^ These findings suggest that RNF213 R4810K directly participates in angiogenesis during the course of MMD through intracellular mechanisms.

Some studies have suggested that the effect of RNF213 R4757K on MMD‐associated angiogenesis may be associated with inflammation.^44^ Hatasu et al. have also shown that interferon beta can increase RNF213 gene expression in HUVECs in a vascular EC‐specific manner through the STATx‐binding site in the promoter region. These researchers discovered that under the influence of interferons or hypoxia, RNF213 R4810K can suppress angiogenesis, whereas wild‐type RNF213 can only inhibit angiogenesis through the interferon signaling pathway.^43^ Kazuhiro et al. demonstrated that the proinflammatory cytokines interferon gamma and tumor necrosis factor‐alpha (TNF‐α) synergistically activate RNF213 transcription through the Akt and protein kinase R pathways. Transcriptome‐wide analysis and subsequent validation also suggested that RNF213 knockdown cells showed reduced proliferative and angiogenic abilities. Moreover, the authors found that RNF213 is involved in the proliferation of ECs by decreasing Akt phosphorylation and inducing the expression of MMP‐1.^45^ These results indicate that RNF213 R4810K can reduce angiogenesis through inflammation.

#### 2.3.2 Growth factors

Vascular endothelial growth factor (VEGF), a key factor in vasculogenesis and angiogenesis, has been widely studied in the context of vascular diseases.^46^ Sakamoto et al. demonstrated that the expression level of VEGF in patients with MMD is four times higher than that in healthy individuals.^47^ In 2012, Park et al. observed that the VEGF‐634G allele has a particularly strong influence on pediatric forms of MMD and is associated with poor collateral vessel formation.^48^ Kang analyzed plasma samples from patients with MMD and healthy controls and found significant upregulation of monocyte chemoattractant protein‐1 (MCP‐1) and VEGF in patients with MMD. Elevated levels of MCP‐1 and VEGF in the plasma of individuals with MMD could potentially contribute to the attraction of vascular progenitor cells and the development of collateral vessels.^49^ Marushima et al. established a chronic cerebral ischemia mouse model in which VEGF‐A was co‐delivered with platelet‐derived growth factor (PDGF)‐BB and reported superior hemodynamic recovery, encephalomyosynangiosis (EMS) collateralization, and ischemic protection. This indicates that VEGF may promote angiogenesis after cerebral ischemia in MMD.^50^ Hiramatsu et al. evaluated the effects of combined gene therapy using VEGF and apelin during indirect vasoreconstructive surgery in a rat model of chronic cerebral hypoperfusion. They reported that VEGF plus apelin therapy during EMS can enhance angiogenesis in rats, which has the potential to become an option for the clinical treatment of MMD.^51^

Transforming growth factor‐beta (TGF‐β) plays a significant role in angiogenesis, cell growth, cell differentiation, and ECM gene expression.^52^ Masato et al. measured the expression levels of TGF‐β1 in the serum and culture medium of SMCs derived from the STA of patients with MMD. They found significantly higher expression levels in both the serum and culture medium than in healthy controls. These results suggest that TGF‐β1 is associated with the pathogenesis of MMD, including the characteristically abundant neovascularization.^53^ Chen has also found upregulation of TGF‐β1 in the middle cerebral artery of patients with MMD, and has shown that TGF‐β1 can upregulate the expression of VEGF to promote angiogenesis in ECs by activating the TGF‐β signaling pathway in vitro.^54^ Wang et al. knocked down RNF213 and found upregulation of TGF‐β1 at both the protein and mRNA levels. They speculated that silencing the RNF213 gene may upregulate TGF‐β1 in bone marrow‐derived mesenchymal stem cells, and this may be related to the pathogenesis of MMD.^55^ Yamamoto et al. found an upregulation of elastin and TGF‐β1 in SMCs derived from patients with MMD and postulated that elastin synthesis and accumulation via the TGF‐β pathway may cause intimal thickening and collateral vessel formation.^56^

PDGF is an important molecule involved in the proliferation, chemotaxis, migration, and survival of ECs.^57^ Aoyagi et al. cultured SMCs derived from the scalp arteries of patients with MMD and have reported both reduced expression of the PDGF receptor (PDGFR) and a decreased response to PDGF.^58^ Kang et al. have reported that the expression levels of PDGF‐BB in the plasma of patients with MMD were higher than those in the plasma of healthy controls.^49^ Yamamoto et al. found that replication and migration decreased after PDGF (PDGF‐BB and PDGF‐AA) stimulation, suggesting a failure in the normal arterial repair process, which may contribute to the intimal thickening observed in MMD.^59^ Marushima et al. inserted the PDGF‐BB gene into the temporalis muscle of an EMS mouse model and have shown that the co‐delivery of VEGF and PDGF‐BB improves functional collateralization in chronic cerebral ischemia.^50^ A recent study explored the role of PDGF‐α in angiogenesis after EMS by inactivating PDGFRα in mice. The results revealed that EMS did not significantly enhance cerebral blood flow (CBF), induce the development of ECs, or achieve complete collagen deposition, even though all three processes were verified in mice with normal PDGFRα expression. This strongly suggests that PDGFRα plays an important role in angiogenesis after EMS in cases of MMD.^60^

Hepatocyte growth factor (HGF) is an angiogenic growth factor with multiple functions, including angiogenesis, the regulation of inflammation, inhibition of fibrosis, and regeneration of tissue.^61^ Nanba et al. measured the HGF levels in the CSF of patients with MMD and evaluated the distribution of HGF and its cellular receptor, c‐Met, in the carotid fork. The HGF level in the CSF was notably elevated compared to that in healthy individuals. Additionally, HGF and c‐Met were widely present in the media and thickened intima of the carotid fork in the patients with MMD but not in the healthy controls.^62^ Yamamoto et al. have reported that HGF can stimulate cell migration in SMCs derived from patients with MMD, suggesting that HGF may be involved in neointimal formation and angiogenesis.^59^ Additionally, Abhinav et al. conducted a study using a multiplex Luminex assay to examine the CSF of patients with MMD. In both MMD subtypes (ischemic and hemorrhagic), 41 significantly elevated molecules were identified, many of which have not been previously reported in MMD. The analysis revealed that IL‐13 and IL‐2 levels were negatively correlated with the preoperative cerebral perfusion status in patients with MMD. Additionally, seven factors were positively correlated with the degree of angiographic revascularization achieved postoperatively.^63^

Basic fibroblast growth factor (bFGF) plays a crucial role in angiogenesis and arteriogenesis by influencing the migration, replication, and maturation of ECs.^64^ Malek et al. analyzed CSF samples collected predominantly from pediatric patients with MMD and healthy controls and found that the bFGF level in the CSF was significantly elevated in patients with MMD. Their study has also revealed a trend of increasing collateral vascularization associated with higher bFGF levels in the CSF. This suggests that bFGF plays a role in angiogenesis during MMD.^65^ bFGF immunoreactivity was also detected in ECs and SMCs from the STA of patients with MMD.^66^ Yamamoto et al. have found that bFGF can stimulate DNA synthesis in SMCs derived from patients with MMD, suggesting that bFGF may promote SMC proliferation and angiogenesis.^59^

### 2.4 Genetic susceptibility in the pathogenesis of moyamoya disease

The literature suggests that genetic factors play an important role in the occurrence and development of MMD^67^ because the disease has a high incidence in specific areas and ethnic groups^68^ and 10% of patients with MMD have familial aggregation.^69^, ^70^

Polymorphisms in susceptibility genes are closely related to the clinical characteristics of patients with MMD, such as the age of onset, initial symptoms, severity, vascular pattern, and prognosis. Therefore, genetic studies are expected to provide a basis for the clinical diagnosis and treatment of patients with MMD (Table 1).

**TABLE 1:** The study of genes associated with moyamoya disease (MMD; except RNF213).

Gene	Study	Ethnic origin	Patients/controls	Study design	Methods	Results
Matrix metalloproteinase (MMP) **and** tissue inhibitors of metalloproteinase (TIMP)	Kang et al. 2006^71^	Korean	66/50	Case–control study focused on single nucleotide polymorphisms (SNPs) in TIMP‐2, TIMP‐4 gene	DNA sequencing technology	G/C heterozygous genotype at position ‐418 in TIMP‐2 promoter could be a genetic predisposing factor for familial MMD
	Paez and Yamamoto 2007^72^	Japanese	48/52	Case–control study focused on SNPs in TIMP‐2 gene	DNA sequencing technology	No significant difference between the SNPs in patients with familial and nonfamilial MMD and controls
	Fujimura et al. 2009^73^	Japanese	16/5	Case–control study focused on serum levels of MMP‐2	Enzyme‐linked immunosorbent assay	Serum MMP‐9 level significantly higher in MMD
	Li et al. 2010^74^	Chinese	208/224	Case–control study focused on polymorphisms in MMP‐2/3/9/13 and TIMP‐2 genes	DNA sequencing technology	SNPs of MMP‐3 promoter region associated with MMD and familial MMD in China
	Roder et al. 2011^75^	European	40/68	Case–control study focused on SNPs in 17 genes including TIMP‐2 between MMD and atherosclerotic disease	DNA sequencing technology	No similar results to those of Kang et al. were found
	Wang et al. 2013^76^	Chinese	96/96	Case–control study focused on SNPs in 4 genes including TIMP‐2 and MMP‐3	DNA sequencing technology	No significant correlation between TIMP‐2 and Chinese Han patients with MMD
	Ma et al. 2015^77^	Chinese	86/86	Case–control study focused on MMP‐3 SNPs and the risk of MMD	DNA sequencing technology	6A allele and 6A/6A MMP‐3 genotype significantly increased the risk of MMD
	Wang et al. 2020^78^	All	4711/8704	Association of genetic variants with MMD	Meta‐analysis	TIMP‐2 rs8179090, MMP‐2 rs243865, and MMP‐3 rs3025058 inversely associated with MMD
	Lu et al. 2021^29^	Chinese	84	Serum MMP‐9 level with hemorrhagic MMD	Enzyme‐linked immunosorbent assay	Serum MMP‐9 level > 1011 ng/mL is an independent risk factor for MMD‐related hemorrhagic stroke
Actin alpha 2 (ACTA2)	Guo et al. 2009^79^	European	20	Linkage analysis and association studies of ACTA2 mutations	DNA sequencing technology	Individuals with ACTA2 gene mutations can develop various vascular diseases, including MMD
	Roder et al. 2010^80^	European	39/68	Case–control study focused on exon mutations in ACTA2 gene	DNA sequencing technology	R179H heterozygous mutation in exon 6 may be associated with MMD
	Shimojima and Yamamoto 2009^81^	Japanese	53	Association study focused on ACTA2 mutations	DNA sequencing technology	ACTA2 is not a major responsible gene for MMD
	Hu et al. 2013^82^	Chinese	55	Association study focused on ACTA2 mutations	DNA sequencing technology	ACTA2 is not a major responsible gene for MMD
**PDGFRB, TGFB1**	Hojo et al. 1998^83^	Japanese	14/10	Expression of TGFB1 in superficial temporal artery (STA) in patients with MMD	Enzyme‐linked immunosorbent assay	Significantly increased
	Roder et al. 2010^84^	European	68/40	Case–control study focused on SNPs in pFGF, cellular retinoic acid‐binding protein (CRABP)1, PDGFRB, TGFB1 genes	DNA sequencing technology	Rs382861 in PDGFRB and rsl800471IN TGFBl may be associated with MMD
	Liu et al. 2012^85^	European	40/68	Further analysis of TGFB1 gene	DNA sequencing technology	No new genetic variants uncovered
	Wang et al. 2013^76^	Chinese	96/96	Case–control study focused on SNPs in four genes including PDGFRB	DNA sequencing technology	No significant association between PDGFRb and MMD
Vascular endothelial growth factor (VEGF)	Park et al. 2012^48^	Korean	107/243	Case–control study focused on SNPs in VEGF, KDR genes	DNA sequencing technology	VEGF‐634G allele associated with pediatric MMD and poor collateral vessel formation
	He et al. 2014^86^	Chinese	53/50	Association study focused on sVEGFR	Enzyme‐linked immunosorbent assay	Lower levels of sVEGFR‐1 and sVEGFR‐2 have favorable surgical prognoses
Human leukocyte antigen (HLA)	Kitahara et al. 1982^87^	Japanese	23	Type i HLA genotyping (HLA A/B/C) and association study	PCR	Association of MMD with HLA A*24, HLA B*46, and HLA B*54 alleles
	Aoyagi et al. 1995^88^	Japanese	32/178	Type i and ii HLA genotyping (HLA A/B/C/DR/DQ) and association study	PCR	Association of MMD with HLA B*51 allele
	Inoue et al. 1997^89^	Japanese	71/525	HLA genotyping (HLA A, DRB1/DQA1/DQB1/DPA1) and association study	PCR‐SSOP	Association of MMD with HLA DRB1*0502, HLA DRBi*0405, and HLA DQBi*0401 alleles
	Han et al. 2003^90^	Korean	28/198	HLA genotyping	ARMS‐PCR	Association of MMD with HLA‐B35 allele
	Hong et al. 2009^91^	Korean	70/207	HLA genotyping	PCR sequence‐specific oligonucleotide hybridization	Association of familial MMD with HLA‐DRB1*1302 and HLA‐DQB1*0609 alleles
	Kraemer et al. 2012^92^	Caucasian	33/database	HLA genotyping and association study	PCR‐SSOP	Strong significant association of MMD with HLA DRB1*03 and HLA DRB1*13 alleles. Weaker significant association of MMD with HLA A*02 and HLA B*08
	Tashiro et al. 2019^93^	Japanese	136/database	HLA genotyping	Next‐generation sequencing	Association of MMD with HLA‐DRB1*04:10
	Jiang et al. 2021^94^	Chinese	775/2031	HLA genotyping	HLA imputation method	Association of MMD with HLA‐DQA2 and HLA‐B
Endothelial NO synthase (eNOS)	Park et al. 2011^95^	Korean	28/7	Association study focused on polymorphisms in eNOS gene (7q36)	DNA sequencing technology	Not significantly associated with MMD but associated with MMD phenotype
**Hcy,** methylenetetrahydrofolate reductase (MTHFR)	Duan et al. 2018^96^	Chinese	1942/5084	Genome‐wide association study with MMD	Genome‐wide association study	Association between rs9651118 in MTHFR and high‐serum homocysteine in MMD
	Park et al. 2014^97^	Korean	20/23	Case–control study focused on SNPs in MTHFR 677C > T and 1298A > C	DNA sequencing technology	Association of pediatric MMD with MTHFR 677CT + TT
**GUCY1A3**	Hervé et al. 2014^98^	European	Three families	Association study focused on GUCY1A3 mutations	Whole‐genome linkage analysis	Association of MMD with GUCY1A3 homozygous mutations
	Wallace et al. 2016^99^	American	96	Association study focused on GUCY1A3 mutations	DNA sequencing technology	Association of MMD with GUCY1A3 homozygous mutations
	Zhang et al. 2017^100^	Chinese	225/300	RNF213, ACTA2, BRCC3, GUCY1A3 genotyping and association study	DNA sequencing technology	No significant association between GUCY1A3 and MMD
**BRCC3**	Miskinyte et al. 2011^101^	American	10	Association study focused on 3 cases of familial moyamoya syndrome (MMS)	DNA sequencing technology	Association of MMD with BRCC3 mutations
	Janczar et al. 2014^102^	American	1	Case report of a SHAM	DNA sequencing technology	Association of MMD with BRCC3 mutations
	Lavin et al. 2016^103^	European	1	Case report of a SHAM	DNA sequencing technology	Association of MMD with BRCC3 mutations
	Zhang et al. 2017^100^	Chinese	225/300	RNF213, ACTA2, BRCC3, GUCY1A3 genotyping and association study	DNA sequencing technology	No significant association between BRCC3 and MMD
	Tzeravini et al. 2022^104^	European			DNA sequencing technology	Association of MMD with BRCC3 mutations
*Drosophila diaphanous* 1 (DIAPH1)	Kundishora et al. 2021^105^	Non‐East Asian	24 + 84	Identification of additional rare, large‐effect variants in the top candidate gene of MMD	DNA sequencing technology	DIAPH1 is a novel risk gene for MMD

#### 2.4.1 RNF213

The RNF213 gene, which is located in the q25.3 region of human chromosome 17,^106^ contains 72 exons and encodes a protein that catalyzes protein depolymerization and ubiquitin linking.^107^ In 2011, Kamada et al. conducted a genome‐wide association study of single nucleotide polymorphisms (SNPs) in 117 patients with MMD. The results showed that 335 SNP loci on chromosome 17q25 are strongly associated with an elevated risk of developing MMD.^106^ In the same year, after comparing patients with familial MMD from different ethnic groups using whole‐genome linkage analysis, Liu et al. found that the p.R4810K point mutation (rs112735431, G → A) in the RING finger domain of RNF213 does not affect the transcriptional and ubiquitin activity of the protein, but could induce MMD pathogenesis, and there was co‐segregation between the RNF213 p.R4810K mutation and the MMD phenotype. The risk of developing MMD in carriers of the mutation is 111.8 times higher than that in noncarriers.^40^ Subsequently, RNF213 has been identified as a susceptibility gene for MMD, and several studies have since focused on the relationship between the RNF213 p.R4810K mutation and MMD.

The p.R4810K mutation in RNF213 is the strongest genetic susceptibility factor in patients with MMD in East Asia. This genetic mutation is present in 80%–90% of patients with familial MMD in Japan and South Korea and 20%–30% of patients with familial MMD in China.^8^ In Japan and Korea, significantly more patients with MMD than individuals from the general population are carriers of this variant (73.44% vs. 1.88% and 82.85% vs. 2.18%, respectively; odds ratio >100).^40^, ^106^, ^108^, ^109^, ^110^ In China, these values are 19.95% and 0.66%, respectively, with an effect value of 37.5 (95% confidence interval: 21.9–64.2).^100^, ^111^ The RNF213 p.R4810K mutation has also been found in patients with MMD in other East Asian countries, but not in the control population.^108^, ^112^, ^113^ However, this mutation is rarely found in White individuals, with a maximum allele frequency of 0.0006.^114^, ^115^ In a study involving whole‐exome sequencing of 125 patients with MMD, the RNF213 mutation was absent in all Caucasian patients. Interestingly, novel variants such as ZXDC (p.P562L) and OBSCN, which are involved in the immune response and muscle development, were identified in Caucasian patients and cases of non‐RNF213 mutations, broadening the genetic understanding of this multiethnic disease.^112^ The studies mentioned above demonstrate that this is a founder mutation in populations with MMD in East Asia and can significantly increase the risk of developing the disease. Although this mutation is present in the general population, most carriers do not present symptoms of MMD, which suggests a low penetrance rate.^116^

The RNF213 p.R4810K mutation can be divided into wild‐type (GG), heterozygous (GA), and homozygous (AA) genotypes. The overall age at onset of patients with the GG genotype is higher than that of patients with the GA genotype. Childhood onset is more common in patients with the AA genotype, and the median age at onset in patients with the GG, GA, and AA genotypes show a gradual downward trend. Furthermore, compared with the GG genotype group, the GA and AA genotypes involve more familial cases.^100^, ^110^, ^117^ Moreover, the age at onset of patients with the AA genotype is lower in cases of familial MMD.^118^ These findings highlight the importance of performing genetic testing in young patients and their relatives. In a cohort of Chinese patients with MMD, the proportion of male patients in the group carrying the mutation was lower than that in the group with the GG genotype. However, two other studies found no correlation between mutations and sex.^100^, ^110^

The clinical symptoms caused by the RNF213 p.R4810K mutation differ in different cohorts, but the mutation group shows a trend toward ischemic symptoms, such as TIA and cerebral infarction, as the first manifestations of the disease. Two Chinese cohort studies on MMD suggest that compared with patients with the GG genotype, those with the mutant genotype are more likely to develop TIA and have less intracranial hemorrhage.^119^, ^120^ Other research results suggest that compared with patients with the GG genotype, those with the GA genotype are more likely to develop ischemia, while compared with patients with the GA genotype, those with the AA genotype are more likely to develop epilepsy.^100^, ^117^ In a cohort of Japanese patients with MMD, cerebral infarction was more common in those with the AA genotype, but the incidence of TIA in these patients was lower than that in patients with the GA genotype. However, there were no differences in the incidence of seizures or intracranial hemorrhage among the three genotypes.^121^ Another Japanese cohort study revealed that TIA was more common in patients with the GA genotype than in those with the GC genotype, and there was no difference in the incidence of cerebral infarction or cerebral hemorrhage among the three genotypes.^110^

In both Chinese and Korean MMD cohorts, the posterior cerebral artery was more likely to be involved in patients with the GA genotype than in those with the GG genotype,^100^ and patients with the GG genotype were more prone to posterior‐to‐anterior pial compensation.^122^ A Japanese study has found that the posterior cerebral artery was more commonly involved in the AA group than in the GA group.^121^ Moreover, a Chinese cohort study has revealed that the GG genotype is associated with enhanced collateral circulation angiogenesis after surgery.^120^

However, the specific functional effects of RNF213 are still being explored. Zebrafish with RNF213 knockdown have shown abnormal neovascularization in the head^40^ and mice deficient in RNF213 have shown ischemia‐induced thinning of the vascular wall intima and media,^123^ suggesting that this gene plays a role in angiogenesis. Although the RNF213 p.R4810K mutation causes local structural changes in the RNF213 protein, the enzymatic properties of the protein are not significantly altered.^124^ Transgenic mice with this point mutation do not exhibit MMD‐like blood vessels,^123^ suggesting that other factors may also play important roles, including epigenetics,^125^ immunity, and inflammation.

In addition to RNF213 p.R4810K, other rare mutations in RNF213 have been found in Asian and Caucasian patients with MMD, including p.M3891V, p.I4076V, and p.V4567M (in the Japanese population)^106^, ^113^; p.A4399T, p.D4013N, p.E4950D, pD4863N, and more than 30 other mutations in the Chinese population^40^, ^100^, ^126^; and p.K4115del, p.S4118F, p.D4013N, and more than 12 other mutations in Western populations. Therefore, p.A4399T may be associated with hemorrhagic MMD. The phenomenon of p.D4013N co‐segregation has been confirmed in European and American families with MMD, and this mutation is strongly suspected to be a founder mutation in White populations.^40^, ^113^, ^127^ A study conducted among a group of European individuals revealed that specific uncommon RNF213 variants—which are located in the C‐terminal region and include the RING finger domain (amino acid numbers 3997–4093)—are linked to MMD in White individuals.^128^ Further studies are required to confirm the role of these rare mutations in MMD.

#### 2.4.2 Tissue inhibitor of metalloproteinase and matrix metalloproteinase genes

MMPs are a large family of proteins that require metal ions as cofactors. Its family members have similar structures, and its main physiological function is to regulate the degradation and remodeling of the ECM. Tissue inhibitors of metalloproteinases (TIMPs) are naturally specific inhibitors of MMPs.^129^ In 2006, Kang et al.^71^ directly sequenced the promoter, exon–intron junction, and exon regions of TIMP‐4 and TIMP‐2 in 11 Korean patients with familial MMD, 50 Korean patients with nonfamilial MMD, and 50 healthy controls. They found that the heterozygous genotype for the GC substitution at site 418 of the TIMP‐2 promoter may be a genetic susceptibility factor for familial MMD. However, in 2007, Paez and Yamamoto conducted a study on the genotype frequency of a specific SNP in seven Japanese individuals with familial MMD, 41 individuals with nonfamilial MMD, and 52 healthy controls,^72^ and found no significant differences in the SNP frequencies between the three groups. Similarly, in 2011, Roder et al.^75^ did not find differences in the frequencies of the SNP rs8179090 C/G in TIMP‐2 between 40 European patients with MMD and 68 healthy controls. In 2013, a study including 96 patients with MMD and 96 healthy controls performed by Wang et al.^76^ has revealed no significant correlation between TIMP‐2 and MMD in Chinese Han patients; however, this finding needs to be verified by further studies owing to the small number of included cases. In 2010, a large cohort study by Li et al.^74^ conducted among 208 patients with MMD and 224 controls has shown that gene polymorphisms in the MMP‐3 promoter region are associated with MMD and familial MMD in China. In 2015 in a Chinese cohort of 86 patients with MMD and 86 healthy controls, Ma et al.^77^ have found that the 6A allele and 6A/6A MMP‐3 genotype significantly increased the risk of MMD. In 2009, Fujimura et al.^73^ have found that serum MMP‐9 levels were significantly higher in patients with MMD than in healthy controls. Additionally, in 2021, Lu et al.^29^ have identified that MMP‐9 acts as a biomarker for predicting hemorrhagic events in MMD, with serum levels >1011 ng/mL being an independent risk factor for MMD‐related hemorrhagic stroke. In 2020, Wang et al.^78^ published a meta‐analysis that evaluated seven polymorphisms in 4711 patients with MMD and 8704 controls. They found a negative correlation between MMD and the TIMP‐2 rs8179090, MMP‐2 rs243865, and MMP‐3 rs3025058 polymorphisms.

In summary, an imbalance in TIMP and MMP levels can lead to abnormal metabolism in the vascular endothelial ECM, resulting in abnormal ECM and SMC dynamics, and ultimately leading to the development of MMD.^49^

#### 2.4.3 Actin alpha 2

The actin alpha 2 (ACTA2) gene encodes tissue‐specific α2 smooth muscle actin. It is mainly expressed in vascular SMCs and provides the basic structural unit for their contraction.^130^ In 2009, Guo et al.^79^ conducted a genetic linkage analysis and genome‐wide association study of 20 patients with ACTA2 mutations, showing that patients with mutations in this gene can develop various vascular diseases (including MMD) and pointing out that the underlying mechanism is vascular SMC migration caused by the ACTA2 SD4 domain (p.R258C/H, P.R212) mutation. In 2010, Roder et al. analyzed six exons of the ACTA2 gene^80^ in 39 patients with MMD and 68 healthy controls. They revealed that the R179H heterozygous mutation in exon 6 may be involved in the pathogenesis of MMD, suggesting that a heterozygous mutation in the ACTA2 gene may increase the risk of developing MMD. However, in 2009, Shimojima and Yamamoto^81^ sequenced all the exons of ACTA2 in 53 Japanese patients with MMD and found no mutations, while in 2013, Hu et al.^82^ directly sequenced nine exons and exon–intron junctions of ACTA2 in 55 Chinese patients with MMD and also found no mutations, suggesting that ACTA2 does not play a major role in the pathogenesis of MMD. Studies with larger sample sizes and further validation among patients with MMD of different ethnicities are required to clarify these contradictory observations.

#### 2.4.4 DIAPH1

The mammalian homolog of *Drosophila diaphanous* 1 (DIAPH1) is a formin protein,^131^ which stimulates the assembly of actin filaments at the barbed ends after activation by Guanosine triphosphate‐bound RhoA.^132^ DIAPH1 promotes the formation of platelets in megakaryocytes through the regulation of actin and the microtubule cytoskeleton,^133^ which can cause macrothrombocytopenia and extend the spectrum of DIAPH1‐related diseases.^134^ In a recent investigation of genetic associations, whole‐gene exon sequencing was employed to detect rare deleterious variants of DIAPH1 in 24 and 84 probands from a discovery and validation group, respectively, all of whom were non‐East Asian individuals with MMD. These variants were linked to thrombocytopenia, clinical manifestations such as ischemic stroke, and angiographic stage.^105^ These findings suggest that DIAPH1 is a novel risk gene for MMD. However, studies on DIAPH1 mutations in other populations are necessary to confirm this conclusion.

#### 2.4.5 Growth factor‐related genes

The protein TGFB1 is involved in regulating cell growth and differentiation and has been found at higher levels in the serum and SMCs of patients with MMD.^83^ PDGF‐B promotes the maturation of mesenchymal cells that have not yet differentiated, and enhances the growth of SMCs in the blood vessels.^135^ In 2010, Roder et al. conducted genotypic sequencing of bFGF, CRABP1, PDGFRB, and TGFBl in 40 patients with MMD and 68 healthy controls. They found that the presence of polymorphisms rs382861 A/C in the PDGFRB promoter region and rsl800471 C/G in exon 1 of TGFBl was associated with MMD.^84^ In a validation trial conducted among 45 Japanese patients with MMD and 79 healthy controls in 2012, Liu et al. did not detect the rs1800471 and rs1800470 polymorphisms previously identified in the European cohort.^85^ The entire exon 1 of TGFB1 was analyzed in a group of 40 European patients with MMD and compared to that of a control group comprising 68 healthy individuals. However, no new disease‐related or genetic variations were identified. In a study conducted in 2013, Wang et al. examined the rs3828610 SNP of PDGFRB in 96 patients with nonfamilial MMD and 96 controls of Chinese Han ethnicity, but did not find a significant association between PDGFRb, MMP3, TIMP‐2, or RNF213 gene polymorphisms and nonfamilial MMD.^76^

VEGF is crucial for the proliferation, migration, and survival of ECs and the regulation of vascular permeability. This imbalance results in poor angiogenesis.^46^ Studies have shown that the VEGF concentration in the plasma of patients with MMD is significantly increased^136^ and that VEGF is highly expressed in the dura mater.^47^ In 2012, Park et al.^48^ performed a study on a cohort of 107 Korean patients with MMD and 243 healthy controls to investigate whether VEGF‐2578, VEGF‐1154, VGEF‐634, VGEF‐936, and receptor‐containing kinase insertion domain (KDR‐604, KDR‐1192, and KDR‐1719) polymorphisms were associated with MMD. The results showed that the frequency of the VEGF‐634 CC genotype was lower in children with MMD, whereas the KDR‐604C/1192A/1719T haplotype increased the risk of MMD development in children. Patients with the VEGF‐634 CC genotype have shown improved postoperative collateral vessel formation. In 2014, He et al.^86^ investigated serum VEGF levels in a cohort of 53 patients with MMD who had undergone indirect bypass surgery and 50 healthy controls. Their findings revealed that soluble VEGF receptor 1 (sVEGFR‐1) and sVEGFR‐2 may be involved in the development of MMD. Furthermore, lower concentrations of sVEGFR‐1 and sVEGFR‐2 were associated with improved collateral circulation 6 months after surgery.

#### 2.4.6 Human leukocyte antigen and its alleles

The human leukocyte antigen (HLA) system is composed of proteins encoded by major histocompatibility complex (MHC) genes that regulate the immune system and distinguish between self and nonself. These genes can be categorized into MHC classes I, II, and III. The primary molecules of classic human MHC Class I antigens include HLA‐A, HLA‐B, and HLA‐C, whereas those of MHC class II antigens include HLA‐DR, HLA‐DQ, and HLA‐DP.^137^ The MHC Class III region is situated between the Class I and II regions and encompasses 55 genes that encode proteins along with five pseudogenes.^138^ In 1982, Kitahara et al.^87^ reported a significant correlation between MDD and HLA‐AW24, BW46, and BW54 for the first time. In 1995, Aoyagi et al.^88^ conducted HLA genotyping in 32 patients with MMD and 178 healthy controls and found a significant positive correlation between MMD and HLA‐B51, whereas the previously described correlations between MMD and HLA‐AW24, HLA‐BW46, and HLA‐BW54 were not confirmed.^87^ In 1997, Inoue et al. performed HLA Class II allele genotyping in 71 Japanese patients with MMD and 525 healthy controls. Their findings revealed a significant correlation between MMD and the presence of the HLA‐DQB1*0502 allele, as well as a notable inverse relationship between MMD and the DRB1*0405 and DQB1*0401 alleles.^89^ In 2003, Han et al.^90^ genotyped the HLA Class I and II alleles of 28 Korean patients with MMD and 198 controls and found a significantly increased frequency of HLA‐B35 in patients with MMD. The frequency of HLA‐B35 was also significantly higher in female patients with tardive MMD. However, the findings reported in the Japanese cohort were not replicated in this study. In 2009, Hong et al. performed a study that involved genotyping HLA Class II alleles in 70 Korean children diagnosed with MMD, including 16 familial cases, and 207 healthy controls. They found that the frequencies of HLA‐DRB1*1302 and DQB1*0609 were significantly elevated in patients with familial MMD, whereas no notable differences were observed in the frequencies of HLA‐DRB1 and HLA‐DQB1 between those with nonfamilial MMD and healthy controls.^91^ In 2012, Kraemer et al.^92^ conducted a comparative study among 33 patients with MMD and healthy controls and found that HLA‐DRB1*03 and HLA‐DRB1*13 were significantly associated with MMD. In 2019, Tashiro et al.^93^ genotyped HLA‐A, HLA‐B, HLA‐C, HLA‐DRB1, HLA‐DQB1, and HLA‐DPB1 in 136 Japanese patients with MDD and found that HLA‐DRB1*04:10 was a risk allele, whereas HLA‐DRB1*04:10‐HLA‐DQB1*04:02 was a risk haplotype for MMD. In 2021, Jiang et al. conducted a study on the MHC region in 755 Chinese individuals with MDD and 2031 healthy controls. They utilized HLA imputation to analyze genetic associations and identified potential predisposing factors for MMD in Han Chinese patients, specifically genetic polymorphisms in HLA‐DQA2 and HLA‐B.^94^

#### 2.4.7 Endothelial nitric oxide synthase gene

Nitric oxide (NO), produced by endothelial NO synthase (eNOS), is an important vascular regulator that reduces vascular tension, inhibits the aggregation of leukocytes and platelets, weakens the chemotaxis of inflammatory mediators, reduces the proliferation of stimulated vascular SMCs,^139^ and protects cerebral nerve cells after ischemic stroke.^140^ In 2011, Park et al.^95^ divided 23 Korean patients with MMD and seven healthy controls into adult and underage subgroups, and further divided the MMD group into ischemic and hemorrhagic groups according to clinical and magnetic resonance imaging (MRI) results. Differences in four eNOS polymorphisms (eNOS‐4A > G, ‐4T > C, 894a < > b, and < > G > T) within and between the groups were evaluated. The results revealed that the frequency of the 4a4b sequence was lower in adults. The distribution of haplotypes (especially A‐4b‐G haplotypes) in patients with MMD differed from that in the control group, and this was particularly obvious in adult patients. Metabolic changes in NO levels caused by eNOS polymorphisms can affect the clinical characteristics of MMD, such as cerebral ischemia and hemorrhage.

#### 2.4.8 Serum homocysteine and its metabolic genes

Homocysteine (a sulfur‐containing amino acid) is an intermediate product of protein metabolism that does not directly participate in protein synthesis. Methylenetetrahydrofolate reductase (MTHFR) is a key enzyme in homocysteine metabolism. It can disrupt blood vessel structure by causing vascular endothelial dysfunction and affecting vascular remodeling.^141^ In 2020, Ge et al.^142^ showed that homocysteine is associated with higher ischemic complication rates in patients with MMD. In 2018, a two‐stage genome‐wide association study was conducted by Duan et al.,^96^ which involved 1492 patients and 5084 healthy controls. The MTHFR gene was found to be associated with abnormal homocysteine metabolism in MMD. The MTHFR 677C > T and 1298A > C polymorphisms are predictive markers of reduced enzyme activity and increased homocysteine levels.^143^, ^144^ In 2014, Park et al. conducted a study in Korea to determine the frequencies of the MTHFR 1298C > T and 12A > C polymorphisms in 20 patients with MMD and 23 healthy controls. The results indicated that the frequency of the MTHFR 677CT + TT genotype was significantly higher in patients with early onset MMD (<10 years), whereas the role of the MTHFR 677C > T and 1298A > C polymorphisms appeared to be limited in this population.^97^ In 2022, He et al.^145^ found that homocysteine levels significantly correlated with poor postoperative angiogenesis in adult patients with MMD.

#### 2.4.9 Guanylate cyclase 1 soluble subunit alpha 3

Guanylate cyclase 1 soluble subunit alpha 3 (GUCY1A3) encodes the α3 subunit of soluble guanylate cyclase, which forms a heterodimeric enzyme with the ß1 subunit encoded by guanylate cyclase 1 soluble subunit beta 3 (GUCY1B3) and is the major receptor for NO.^146^ In 2014, GUCY1A3 mutations were first detected by Hervé et al.^98^ in patients with familial MMD and achalasia. This syndrome was associated with homozygous mutations in GUCY1A3 in all three families included in this study. In 2016, Wallace et al.^99^ sequenced GUCY1A in 96 cases of MMD from unrelated families. Two individuals from unrelated families carried compound heterozygous mutations in GUCY1A3. However, in 2017, Zhang et al.^100^ compared the frequencies of GUCY1A3 genotypes and alleles in 255 Chinese patients with MMD and 300 controls and found no differences between the groups.

#### 2.4.10 Subunit 3 of the BRCA1/BRCA2‐containing complex

Subunit 3 of the BRCA1/BRCA2‐containing complex (BRCC3) is a ubiquitously expressed K63‐linked deubiquitinating enzyme. Knockout of BRACC3 in zebrafish results in angiogenesis defects. In 2011, Myskinite et al.^101^ first studied three unrelated families with X‐linked MMS—characterized by the association of moyamoya angiopathy, short stature, stereotyped facial dysmorphism, and other endocrinopathy symptoms—and showed it to be caused by microdeletions within Xq28 involving several genes, including BRCC3. In 2014, Janczar et al.^102^ reported a case of a child with severe hemophilia A and MDD which led to ischemic stroke. Next‐generation sequencing revealed a large Xq28 deletion encompassing BRCC3 and exons 1–6 of the factor 8 (F8) gene. In 2016, Lavin et al.^103^ reported an adult case of X‐linked MMS and severe hemophilia A. Sequencing results confirmed the contiguous deletion of F8 exons 1–14 together with genes immediately upstream of F8, including BRCC3. In 2022, Tzeravini et al.^104^ reported a case of a 19‐year‐old male patient with hemophilia A and ischemic stroke. Molecular genotyping revealed Xq28 microdeletions encompassing F8 and BRCC3, which are responsible for the final phenotypic characteristics, including stunting and hypergonadotropin hypogonadism. However, in 2017, Zhang et al.^100^ compared the frequencies of BRCC3 genotypes and alleles in 255 Chinese patients with MMD and 300 healthy controls and found no differences between the two groups.

### 2.5 DNA methylation in moyamoya disease

**FIGURE 2:**
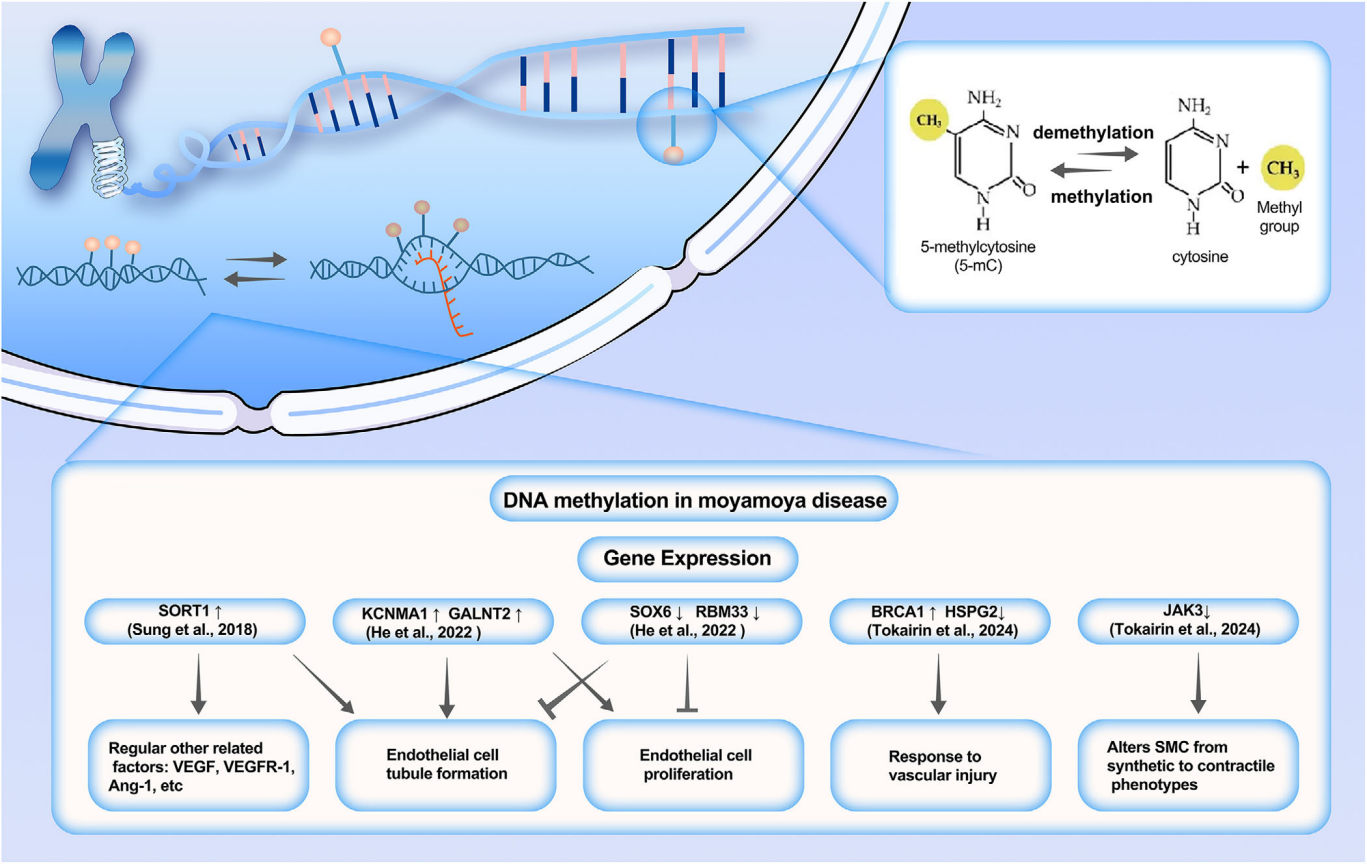
Overview of DNA methylation involved in moyamoya disease. In the nucleus, under the action of DNA methyltransferases, a methyl group is covalently bound to the 5′ carbon of the cytosine in CpG dinucleotides. The level of DNA methylation gives rise to changes in the expression levels of certain genes in moyamoya disease. This has an impact on certain processes related to vascular, such as endothelial tube formation, endothelial cell proliferation, vascular injury response, and so on. BRCA1, breast cancer type 1 susceptibility protein; GACNT2, GalNAc‐type O‐glycosylation and its initiating GalNAc transferase 2; HSPG2, heparan sulfate proteoglycan 2; JAK3, Janus kinase 3; KCNMA1, potassium large conductance calcium‐activated channel subfamily M alpha member 1; RBM33, RNA‐binding protein 33; SORT1, Sortilin 1; SOX6, SRY‐box transcription factor 6.

Epigenetics refers to hereditary structural and biochemical changes in chromatin, without altering DNA sequence, and currently mainly includes DNA methylation, histone modification, and noncoding RNA.^147^, ^148^ DNA methylation is a recognized epigenetic process that entails the addition of a methyl group to the 5′‐cytosine (C) base within a CpG dinucleotide, thereby affecting gene transcription in reaction to environmental influences.^149^ What stands out most is that abnormal DNA methylation patterns are a common molecular feature of vascular diseases, and the pathological patterns seem to be similar.^150^, ^151^, ^152^ In recent years, in MMD, epigenetics, especially DNA methylation, is recognized for its pivotal role in MMD pathogenesis (Figure 2).^153^

Sung et al. analyzed the gene expression profile and DNA methylation profile of endothelial colony‐forming cells (ECFCs) from three MMD patients and two healthy individuals, identifying five candidate genes.^154^ Then, the findings were validated using a dataset of ECFC samples from nine MMD patients and 10 controls. In the end, they found that all five genes were upregulated due to low methylation of specific promoter CpG sites. Furthermore, overexpression of Sortilin 1 (SORT1) inhibited the formation of endothelial tubes and affected various angiogenesis‐related factors. He et al. used the Illumina 850K chip to perform whole‐genome DNA methylation analysis on 10 patients with ischemic smoke disease and 10 healthy individuals.^155^ They found that the genetically methylated level in adults with ischemic MMD was higher than that of healthy individuals. The high methylation regions are mainly concentrated in the intergenic regions. They identified 759 differentially expressed genes and explored the relationship between these genes and MMD through in vitro experiments. The results suggest that low expression of SOX6 and RBM33 may be associated with vascular occlusion in MMD, while overexpression of KCNMA1 and GALNT2 may be associated with angiogenesis. Tokairin et al. used the Illumina 850K methylation chip to profile the whole genome DNA methylation of patients with MMD.^156^ What is noteworthy is that this study was conducted on two separate female cohorts: a non‐Asian cohort (13 MMD patients and seven healthy controls) and an Asian cohort (14 MMD patients and three healthy controls). The results showed that there was significantly lower DNA methylation variability between MMD patients and healthy controls in both MMD female cohorts. Two different ethnic groups showed similar decreases in methylation variability. Subsequent analyses indicated that aberrant methylation genes were primarily associated with methylation and transcription, DNA repair, cytoskeletal remodeling, natural killer cell signaling, cell growth, and migration. The decreased methylation variability in MMD may impede patients' adaptation to environmental changes, thereby disrupting vascular homeostat.

Although the number of studies on DNA methylation in MMD is limited, all of these studies have found crucial genes for the mechanism of MMD in patients compared with healthy controls. Although the DNA methylation profiles of MMD obtained through different methods by various experiments are different, it is difficult to deny that epigenetics, represented by DNA methylation, will be a new direction for unraveling the pathogenesis of MMD.

### 2.6 Inflammatory, immune, and other factors associated with moyamoya disease

The dual‐hit theory of MMD suggests that it is caused by both genetic and environmental factors, implying that it may be a multifactorial disease. This may explain the low expression rate of and regional differences in RNF2 13 Arg4810Lys in MMD.^8^, ^157^ Currently, patients with cerebrovascular characteristics of MMD who also have other factors, such as brain tumors, radiation, and autoimmune diseases, are diagnosed with MMS rather than MMD.^9^ However, the relationship between MMD and MMS has been subject to debate. Given the unclear pathogenesis, it is imperative to discuss and consider the role of external factors to acquire a comprehensive understanding of MMD.

#### 2.6.1 Evidence linking moyamoya disease to immunity and inflammation

**FIGURE 3:**
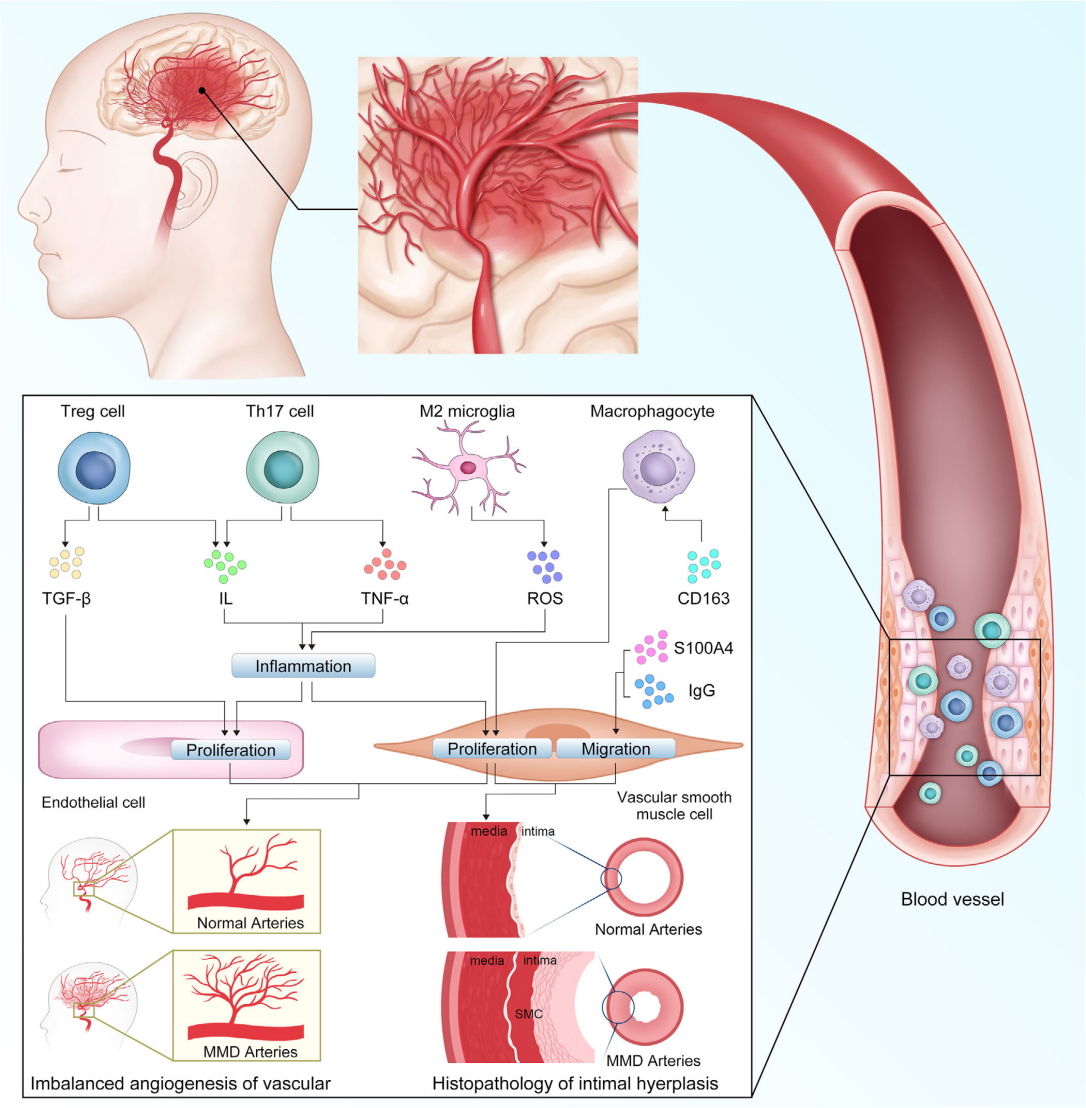
Mechanism of immune infiltration in moyamoya disease (MMD). In moyamoya disease, immune cells and cytokines exert an effect on the vascular endothelial cells and smooth muscle cells. Treg cells secrete TGF‐β and IL‐10. Th17 cells secrete IL‐17 and TNF‐α.M2 microglia secrete ROS. CD163 promotes the regulation of macrophagocyte for VSMC proliferation. Various cytokines directly or indirectly modulate the proliferation and migration of vascular endothelial cells and smooth muscle cells, which may be connect with intimal thickening and vascular compensatory hyperplasia in MMD. IL, interleukin; ROS, reactive oxygen species; TGF‐β, transforming growth factor‐beta; TNF‐α, tumor necrosis factor‐alpha; Treg cell, regulatory T cell; VSMC, vascular smooth muscle cell.

Recent studies have examined the roles of immune and inflammatory mediators in the development of MMD (Figure 3). Junichi et al. were the first to report the association between immunity and MMD. In MMD, an abnormal increase in proliferative SMCs, macrophages, and T‐cells has been detected in thickened vascular walls.^158^ These immune and inflammatory factors appear to be involved in the proliferative transformation of SMCs. One study analyzed histopathological and immunohistochemical findings from the intracranial vessels of patients with MMD, revealing that the internal elastic lamina is significantly distorted and layered, with vacuolar degeneration in the cerebrovascular SMCs. Additionally, immunohistochemical analysis has indicated that SMCs migrate into the thickened intima and exhibit abnormal expression of IgG and S100A4. This suggests that the deposition of these immune‐related factors facilitates SMC migration into the intima through the internal elastic lamina, which leads to intimal thickening and stenosis of the intracranial arteries.^159^ A recent study used high‐density protein arrays to analyze antibodies in the serum of patients with MMD and compared them with healthy control groups. They identified 165 significantly elevated (*p* < 0.05) autoantibodies in MMD, of which six were closely related to MMD. This provides new theoretical evidence for the immune characteristics of MMD.^160^ Fujimura et al. have discovered that the serum levels of soluble CD163 are significantly higher in patients with MMD than in healthy controls. Soluble CD163 serves as an activating marker of CD163+ M2‐polarized macrophages, which play a role in various autoimmune disorders.^161^ They have also found that the effect was most pronounced in cases of Suzuki stage III, which has the most extensive smoke‐like vascular network. In atherosclerosis, CD163+ macrophages promote intraplaque angiogenesis.^162^ CD163 macrophages mediate multiple signaling pathways in various vascular diseases, playing a role in angiogenesis and vascular stabilization.^163^ Similarly, the proangiogenic effect of CD163 may be one of the factors promoting angiogenesis in MMD. Additionally, there is an increase in the expression of stromal cell‐derived factor‐1 alpha in patients with MMD. This factor interacts with its receptor, CXCR4, to regulate the migration of CD34+ cells or EPCs from the bone marrow to the peripheral blood and modulates angiogenesis. This suggests that inflammation may also play a role in the development of MMD.^164^ A prospective cohort study on MMD found that patients with more smoke‐like vessels had higher numbers of CD34+ cells than did the control group and patients without smoke‐like vessels.^165^ CD34+ cells are strongly associated with the formation of new blood vessels in ischemic brain tissue. Furthermore, Weng et al. have demonstrated that the levels of regulatory T cells (Tregs), T helper 17 (Th17), and some of their related inflammatory cytokines—including Treg‐related TGF‐β and interleukin (IL)‐10 and Th17‐related IL‐17, TNF‐α, IL‐6, and IL‐23—are significantly elevated in patients with MMD compared with those with atherothrombotic stroke and healthy controls. However, Tregs from patients with MMD exhibit deficient suppressive functions, leading to a functional imbalance in the Treg/Th17 ratio, which may contribute to the pathophysiology of MMD.^166^ After immune enhancers were administered to RNF213‐deficient mice, MMD was not induced. However, a significant decrease in the proportion of Tregs was found.^167^ This suggests that the differentiation of Tregs may depend on the normal function of RNF213, which may, therefore, regulate the immune tolerance of patients with MMD. In addition to their immune function, the role of Tregs in vascular physiology has been gradually reported in recent years. Tregs regulate the proliferation and apoptosis of ECs through TNFR1, DDL4, and Notch, which are crucial for the stability of angiogenesis.^168^ The special role of Tregs links immunity with MMD and provides a new understanding of the immune pathology of this disease. Chronic inflammation is a fundamental pathological feature of many diseases. A retrospective study by Juan et al. has revealed a strong epidemiological association between MMD and inflammatory diseases such as systemic lupus erythematosus, rheumatoid arthritis, and scleroderma.^169^, ^170^ Noritoshi et al. have identified 338 upregulated genes and 262 downregulated genes in the microglia of blood cultures of patients with MMD.^171^ M2 microglia can promote the production of reactive oxygen species (ROS) and inflammation by inhibiting RNF213 and suppressing phagocytosis. This inflammation in the blood vessels leads to the proliferation of vascular SMCs and ECs.

Duan et al. conducted the first comprehensive and in‐depth proteomic analysis of exosomal proteins in patients with hemorrhagic MMD, and found that exosomal proteins could induce mitochondrial dysfunction and increase ROS levels in the vascular ECs of this patient population.^35^ They suggested that exosomes may promote the occurrence of cerebral hemorrhage in MMD by damaging ECs. Meanwhile, ROS is one of the upstream initiators of inflammation, which is crucial for the persistence of chronic neuroinflammation.^172^ Huang et al. performed high‐throughput sequencing to obtain a comprehensive miRNA expression profile of exosomes in the plasma of patients with MMD and identified 1002 differentially expressed miRNAs. Functional analysis showed that they were mainly enriched in axon guidance, actin cytoskeleton regulation, and MAPK signaling pathways.^173^ The MAPK signaling pathway, an important signaling pathway in inflammation, is reportedly involved in the pathogenesis of MMD.^174^ However, miRNAs in exosomes may not fully reflect the cellular characteristics. Recent research on exosomes from leukocytes in patients with MMD has revealed that there are significant differences between the exosomes of healthy controls and those of patients with MMD, as well as between the exosomes from different subtypes of MMD.^175^ This suggests that exosomes play an indispensable role in the pathogenesis of MMD, both within and outside cells. Apart from the MAPK signaling pathway, numerous other signaling pathways play key roles in MMD. NF‐κB, an important regulator of upstream signaling pathways involved in the inflammatory response, has been widely found in intracranial aneurysm, atherosclerosis, and other diseases.^176^, ^177^, ^178^ Midori et al. have reported that in MMD, inhibiting the ubiquitin ligase activity of RNF213 enhances the activation of NF‐κB, which ultimately leads to cell apoptosis.^179^ After conducting in vitro experiments, they ultimately concluded that this RNF213‐dependent regulation mode of NF‐κB activity affects cell apoptosis in MMD. Although the authors did not conduct experiments using vascular cells, this finding provides a new direction for determining the role of RNF213 in MMD. Yifan et al. have identified the potential of ACAN, FREM1, TOP2A, and UCHL1 as diagnostic markers in MMD through machine learning.^180^ Among them, ACAN has a relatively obvious positive correlation with the JAK/STAT signaling pathway. However, they did not delve deeply into the impact of the JAK/STAT signaling pathway on MMD. Currently, research mainly focuses on the role of the JAK/STAT signaling pathway in inflammatory diseases and tumors.^181^ However, its role in regulating cell apoptosis, proliferation, and inflammation makes it a promising area of research in vascular diseases. In the future, the association between the JAK/STAT signaling pathway and MMD may yield significant conclusions.

Inflammatory and immune factors are primarily associated with the promotion of cell proliferation and angiogenesis. Normal angiogenesis is essential for tissue regeneration in the human body, but imbalanced angiogenesis is also an important pathological process in many vascular diseases, including MMD. Additionally, considerable molecular and clinical evidence suggests that mechanisms related to the immune response may trigger a second attack of MMD. Therefore, inflammation and immunity alone may not initiate the pathological process, but rather work together with genes to complete the pathological process of vascular narrowing and proliferation.

#### 2.6.2 Postradiation moyamoya syndrome

Radiation therapy is a milestone in cancer treatment; however, it causes unavoidable damage to the vasculature surrounding the tumor, especially the arteries. This damage is not only related to the radiation dose but also to the target vessel, tumor type, patient's age, and inflammation.^182^ A case series and literature review conducted by Marcello et al. among children with brain tumors revealed that those under the age of 5 years who receive high doses of radiation and have neurofibromatosis type 1 are more prone to developing postradiation MMS. In the follow‐up after the MMS diagnosis, the Suzuki score of these patients was mostly maintained at II–III.^183^ Long‐term radiation‐induced vascular pathological changes include endothelial hyperplasia, basement membrane thickening, adventitial fibrosis, and vessel dilatation.^184^, ^185^ In a case report of radiation‐induced MMS after childhood astrocytoma, vascular biopsy revealed thickening of the vessel walls, absence of the inner elastic layer, and sclerosis. Similar pathological changes in the vessel walls and intravascular elastic lamina have been observed in patients with MMD. However, hyaline degeneration appears to be caused by radiation damage rather than MMD.^186^ Furthermore, whether the characteristic changes in this vascular injury are caused by radiation or tumors is still unclear.

Most cases of radiation‐induced MMS have been reported in case reports, and in‐depth cohort studies are lacking. Therefore, it cannot be assumed that radiation‐induced MMS is associated with the same pathological changes as is MMD.^183^, ^187^ The evolutionary process of MMS after radiotherapy is similar to that of MMD, although the vascular pathological changes differ. This provides pathological evidence and theoretical support for the differential diagnosis and treatment of radiation‐related MMS and MMD.

#### 2.6.3 Postinfectious moyamoya syndrome

Infection is a common cause of vascular damage leading to MMS. A series of retrospective studies has revealed that AIDS (50%), childhood status (66.6%), and female sex (55.56%) are high‐risk factors for postinfectious MMS.^188^ Among these, patients with AIDS are more likely to develop MMS, which may be related to the side effects of long‐term antiretroviral drug use.^189^ However, whether these patients had MMD before infection is difficult to determine. Infection may not be the cause but rather a trigger. Fever, headache, and other symptoms caused by infection may prompt asymptomatic patients with MMD to undergo relevant checks. Therefore, infection may increase the detection rate of asymptomatic MMD. In one study, *Propionibacterium acnes* was bilaterally injected into the carotid bifurcation of rats. After several weeks, the authors observed that the inner elastic lamina of the intracranial internal carotid segment was disrupted.^190^ However, no similar phenomenon was observed in rats injected with *Propionibacterium acnes* in the peritoneal cavity or occipital region. Therefore, it cannot be ruled out that the changes in the cervical internal carotid artery are caused by the injected trauma factor. Moreover, no progressive vascular occlusion or smoke‐like vessels that are characteristic of MMD were observed in the rats. Several studies have shown that RNF213 is involved in antibacterial and immune regulatory processes.^40^, ^146^ Therefore, RNF213 is likely a key factor linking infection to MMS. However, currently, there are no mouse model experiments on the dual hit of genes and infection. Therefore, infection may be either a triggering factor for the symptoms or a driving factor in the pathogenesis of MMD.

#### 2.6.4 Other nonhereditary factors

In a retrospective study, Yohei et al. collected detailed information on 93 patients with unilateral MMD, including demographic characteristics, RNF213 R4810K mutation, lifestyle factors (such as smoking and alcohol consumption), past medical history, and angiographic findings.^191^ They found that alcohol consumption, but not smoking, is significantly associated with progression on the contralateral side. Recurrent stroke is a common symptom of MMD, while smoking, alcohol consumption, and obesity are common risk factors for stroke, especially in older patients.^192^, ^193^ Therefore, lifestyle management is an important measure to assist patients with MMD in maintaining brain health. Moreover, Xiaofan et al. conducted a study among 60 patients with MMD and reported that the balance of gut microbiota (detected through stool samples) is related to MMD.^194^ In patients with MMD, the abundance of *Clostridium* and *Fusobacterium* species was increased in the intestine, whereas the abundance of *Bifidobacterium* and *Enterobacter* species was decreased.

### 2.7 Exploring the pathogenesis of moyamoya disease using comprehensive multiomics

**FIGURE 4:**
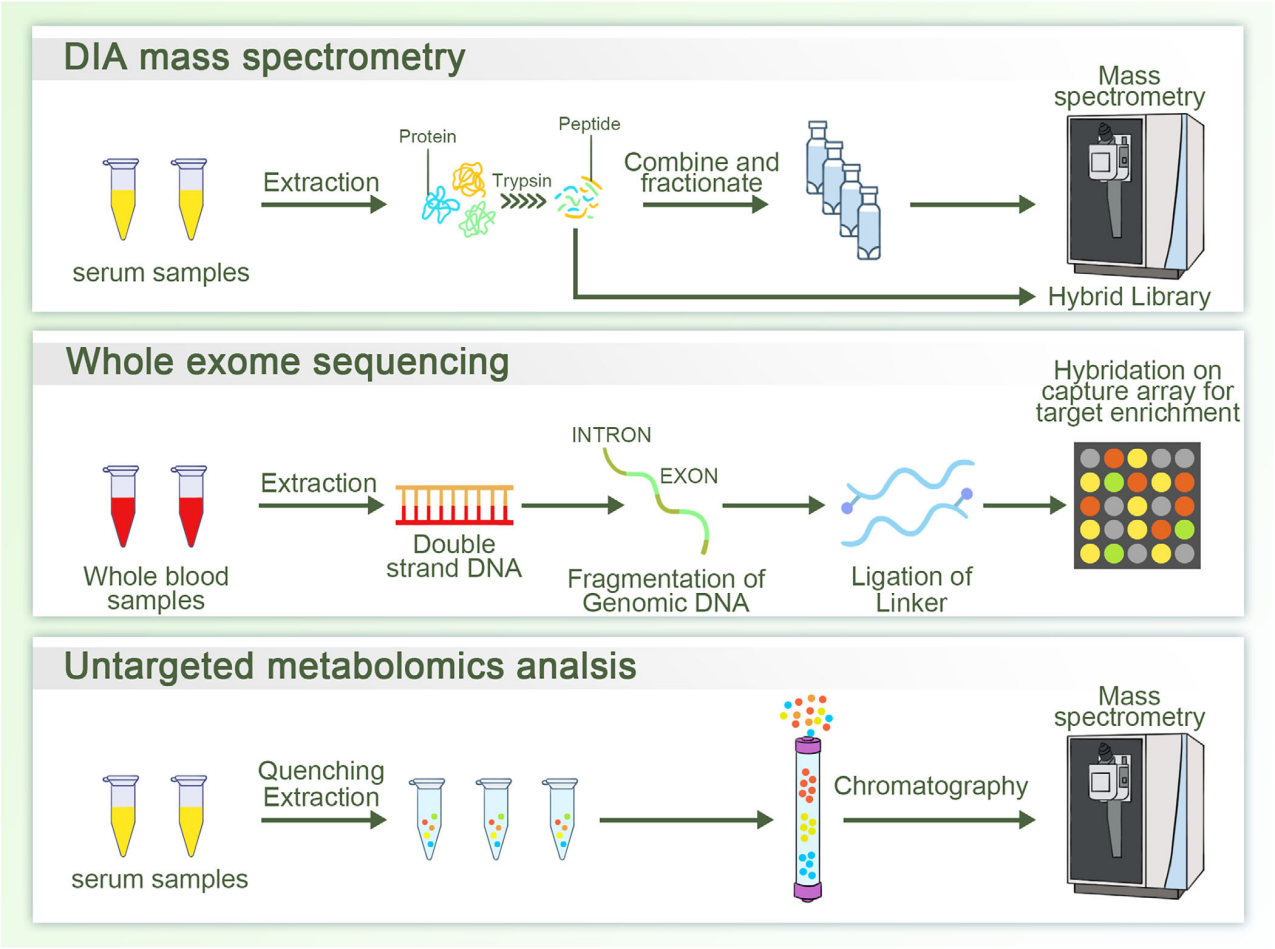
Multiomics in the moyamoya disease. The figure illustrates the multiomics approach to moyamoya disease. DIA mass spectrometry: Proteins are extracted from serum samples. After undergoing treatment with trypsin, proteins are transformed into peptide segments. Following binding and fractionation, mass spectrometry analysis is carried out. Whole‐exome sequencing: Double‐stranded DNA is extracted from whole blood samples. DNA is cut by the ligation‐based method to obtain exons. The capture array hybridization is employed for target enrichment analysis; Untargeted metabolomics analysis: Metabolites are extracted from serum samples. The samples are introduced into the chromatography system and undergo mass spectrometry analysis. DIA, data‐independent acquisition mass spectrometry.

The results of previous studies suggest that the pathogenesis of MMD is driven by a complex system of interactions among multiple factors at different levels, encompassing genes to proteins, intracellular to extracellular dynamics, and cells to tissues and organs. Therefore, studying the pathogenesis of MMD requires multiomic research—including genomics, epigenetics, transcriptomics, proteomics, and metabolomics—which is changing our understanding of cell biology in MMD.^195^ He et al. have conducted several meaningful studies of the proteins, exosomes, and metabolism of MMD using multiomic methods (Figure 4).^34^, ^196^, ^197^ Yasuhisa et al. analyzed the relationships between 11 virus‐related factors that could be associated with cerebrovascular disease and the pathogenesis of MMD through multiomics. These results revealed that the 11 investigated viruses were unlikely to have an impact on the development of MMD. However, the use of multiomics to create a systematic map of viral infections in patients with MMD has provided crucial evidence for exploring whether MMD is a type of immunovascular disease.^198^ Huiqin et al. analyzed the peripheral blood of patients with MMD and found that the expression of granulocyte‐macrophage colony‐stimulating factor (GM‐CSF), originating from Th1 and Th17 cells, was significantly elevated. These multiomic results provide the first evidence of the differential expression of GM‐CSF in MMD and suggest that it may participate in the formation of abnormal vascular networks in MMD.^199^

In summary, multiomic research requires in‐depth integration of data to overcome the limitations of single‐omic research. In MMD, multiomics facilitates building a comprehensive regulatory network of gene expression, which helps to elucidate the regulation and causal relationships between various factors and promotes the discovery of new MMD biomarkers.

### 2.8 Advanced experimental models in moyamoya disease research

As more research is being conducted on genes related to MMD, the connection between these genes and the development of MMD requires additional exploration using modern experimental models and techniques. According to genetic mutation‐related theories, Shinya et al. attempted to knock down RNF213 in mice, and RNF213(−/−) mice did not develop MMD spontaneously. However, the endometrium and middle layer were significantly thinner in RNF213(−/−) mice. Kaku et al. have reported that the outer diameter of the vasculature narrows before the inner diameter in MMD. Therefore, RNF213 may be an early promoting factor of MMD.^200^ Thus, the absence of the RNF213 gene in mice did not lead to the spontaneous development of MMD, suggesting that a loss of function in RNF213 was not sufficient to trigger MMD.^201^ Moreover, knockdown of RNF213 in zebrafish results in vessel wall abnormalities in both main arteries as well as neovascularization. However, the typical pathological features of MMD were not observed.^202^ Various mutation sites of RNF213 in MMD exist, and new mutations are continuously being discovered; however,^40^ it is unclear whether these mutations enhance or weaken the function of RNF213. Limited by the current gene technology and our understanding of RNF213, simple knockout of RNF213 or a specific site of RNF213 in animal models may not fully simulate the genetic state in vivo. Therefore, successful cases of RNF213‐based animal models of MMD have not yet been reported. In a study conducted by Ichiro et al., immunoembolic material was injected into the internal carotid artery of mice to induce a local immune response. Thickening of the elastic layer of the vascular intima was observed in the vascular sections. After treatment, carotid angiography revealed stenosis at the end of the internal carotid artery and collateral vessels in the basal brain region.^203^ However, this study did not identify pathological alterations in the circle of Willis similar to those in MMD. This may be attributed to incomplete vascular occlusion. Additionally, differences in hemodynamics and cellular physiology between humans and mice pose obstacles to the construction of animal models. Hence, there is an urgent need for an experimental model that is more consistent with human physiological characteristics.

Kikutaro et al. used iPSCs to induce differentiation into vascular SMCs and ECs in MMD for the first time. They compared the transcriptomic features of vascular SMCs and ECs between healthy controls and patients with MMD.^204^ They found that the SMCs in the MMD group did not differ significantly from those of the control group. This suggests that the pathological changes in the SMCs in MMD may be driven by ECs or other factors. However, owing to the lack of SMCs from the human vasculature as a control, it is difficult to determine whether SMCs induced by iPSC can display the characteristics of in vivo conditions. Blecharz et al. established a cell model to explore the role of VEGF‐2 in MMD. They used the serum of patients with MMD to culture cerebral ECs in vitro. In the in vitro culture, compared to the control group, the serum of patients with MMD caused the cerebral ECs to exhibit characteristics of MMD in vivo, including an increased secretion of angiopoietin‐2 and decreased endothelial integrity.^205^ However, experimental evidence regarding low expression of angiopoietin‐2 is lacking. Furthermore, the detailed mechanisms and signaling pathways through which angiopoietin‐2 affects ECs have not yet been explored. Based on a cell model cultured with serum, we found that FLNA and ZYX are related to the pathological mechanism of intimal hyperplasia in MMD.^30^ FLNA and ZYX promote the proliferation of ECs that overexpress the FA signaling pathway, thereby driving the thickening of the vascular intima in MMD.

Wimmer et al. used hPSCs derived from patients with diabetes to generate human vascular organoids that exhibited morphological, functional, and molecular characteristics of diabetes. By transplanting human vascular organoids into immunocompromised mice, a stable, perfused vascular tree was formed that simulated the microvascular changes observed in patients with diabetes.^206^ Vascular organoids are emerging as a promising experimental model for overcoming research barriers in vascular disease studies. They provide a new experimental basis for studying the pathogenesis and treatment of cerebrovascular diseases by combining multiomics, CRISPR, and drug screening. Considering the failure of animal models and the limitations of cell models, a new experimental model that can more comprehensively simulate the pathological features of MMD should be developed. The biological carriers of traditional models may have limited the success of animal models of MMD. However, it is difficult for cell models to reflect pathological features at the tissue and organ levels. Organoids are excellent experimental models for other diseases. He et al. pioneered the induction of vascular organoids derived from the iPSCs of patients with MMD and controls. Using single‐cell sequencing and proteomics, they discovered the crucial roles of TUBA and TUBB in the transformation, proliferation, and migration of VSMCs. Their findings provide the latest pathological evidence at the organoid level regarding how VSMCs cause intimal hyperplasia in MMD.^207^ In the future, cerebral organoids may be essential in advancing research regarding the pathogenesis of MMD.

## 3 IMAGING DIAGNOSIS

In 1997, the Research Committee on Spontaneous Occlusion of the Circle of Willis (Moyamoya Disease) released guidelines for the diagnosis and treatment of MMD.^208^ Accordingly, a definitive diagnosis of MMD is made in patients who exhibit bilateral stenosis or occlusion at the terminal segments of the intracranial internal carotid artery or proximal areas of the anterior and/or middle cerebral arteries, along with the bilateral formation of abnormal vascular networks near these occlusive or stenotic sites during the arterial phase. The diagnostic criteria for MMD remained unchanged in the updated guidelines released by the Research Committee on the Pathology and Treatment of Spontaneous Occlusion of the Circle of Willis in 2012.^209^ At present, MMD can be diagnosed using either cerebral angiography or MRI/magnetic resonance angiography (MRA; Figure 3).

### 3.1 Cerebral angiography

Cerebral angiography provides notable benefits in illustrating the anatomical features of both major and minor cerebral vessels, making it the gold standard method for diagnosing MMD. A complete angiographic examination should include a thorough study of five or six key vessels: both external carotid arteries, both internal carotid arteries, and one or two vertebral arteries based on the observed collateral patterns. Suzuki et al. classified the progression of MMD into six phases according to dynamic changes in the affected vessels, a system that is still relevant today. Phase 1 marks the onset of narrowing at the bifurcation of the internal carotid artery and at the carotid fork; Phase 2 involves visible moyamoya collaterals around these narrowed areas; Phase 3 indicates worsening conditions with the increased presence of moyamoya collaterals; Phase 4 shows further deterioration in the narrowed vasculature while moyamoya collaterals start to diminish; Phase 5 is characterized by the occlusion of larger related vasculature along with a more pronounced reduction in surrounding moyamoya alterations; and Phase 6 sees a total loss of moyamoya collaterals and internal carotid artery system vessels, with blood supply to the internal carotid artery territories being sourced from the external carotid arteries instead.^210^

### 3.2 MRI and MRA

The increasing accessibility of MRI and MRA has resulted in a growing preference for these techniques as primary imaging methods when patients with MMD exhibit neurological symptoms. Nonetheless, the effectiveness of MRA is substantially influenced by the strength of the static magnetic field. The most recent recommendations from the Research Committee for the Diagnosis of Moyamoya Disease suggest utilizing an MRA machine with a magnetic field strength of 1.5 T, while using machines with magnetic field strengths of 0.5 T or 1.0 T is not advised.^209^

Diffusion‐weighted imaging is superior in identifying acute infarcts, whereas T1‐ and T2‐weighted imaging are more effective in detecting chronic infarcts. T1‐weighted MRI shows dilated moyamoya vessels in the basal ganglia and thalamus. The most characteristic feature of MMD on MRI is reduced flow voids in the internal, middle, and anterior cerebral arteries accompanied by prominent flow voids in the basal ganglia and thalamus resulting from collateral vessels related to moyamoya. These observations are nearly definitive for diagnosing MMD.^211^

MRA is a valuable noninvasive tool for diagnosing MMD. It can accurately detect stenotic lesions in the distal portions of the carotid arteries. Furthermore, MRA has simplified the identification of asymptomatic individuals with familial MMD. However, it is important to consider the potential for overestimating accuracy owing to variations in imaging quality.^212^, ^213^ MRA can also be used to detect moyamoya vessels surrounding the basal ganglia and thalamus.^213^ Houkin et al. proposed a new grading system for MRA in MMD, in which scores are assigned based on the extent of occlusive changes observed in the internal carotid artery as well as in the horizontal segments of the middle cerebral artery, anterior cerebral artery, and posterior cerebral artery. The MRA score was strongly correlated with the Suzuki score used for cerebral angiography, exhibiting high sensitivity and specificity.^214^

In addition to the previously mentioned methods, several new imaging techniques can be utilized to diagnose MMD. Decreased cortical blood flow linked to MMD can be suggested by fluid‐attenuated inversion recovery sequences that show linear high signals in a sulcal pattern, often known as the “ivy sign.”^215^ High‐resolution imaging of the vessel walls shows concentric enhancement in the distal internal carotid arteries and a decrease in the size of the middle cerebral artery in patients with MMD, regardless of symptom presence.^216^ In 2017, Byeong et al. conducted a study that compared 7.0 T and 3.0 T MRI in patients with MMD, and concluded that there were no statistically significant differences in the assessed parameters, including the Suzuki score, internal carotid artery diameter, and ivy sign score.^217^ However, when compared to 3.0 T MRI, 7.0 T MRI appears to provide a more accurate depiction of the abnormal vascular networks within the basal ganglia. Moreover, four‐dimensional pseudocontinuous arterial spin labeling (ASL) enables direct visualization of the leptomeningeal collateral vessels in cases of MMD.^218^ These neuroimaging techniques can be used to detect different types of collateral vessels (i.e., anastomoses). The phrase “periventricular anastomosis” describes the link between the perforating or choroidal arteries and the medullary artery within the periventricular area.^219^ Choroidal anastomosis is a predictor of rebleeding in patients with hemorrhagic MMD.^220^ A study involving 122 individuals diagnosed with MMD or MMS revealed that sliding‐thin‐slab maximum intensity projection is effective in detecting periventricular anastomosis.^220^ An increasing number of high‐precision MRI techniques will provide greater advantages for the diagnosis of MMD.

### 3.3 Hemodynamics

MMD is angiographically defined based on gradual narrowing or blockage of the terminal internal carotid arteries. This condition is frequently associated with the formation of collateral networks that appear to offer an alternative pathway for CBF. However, this compensatory mechanism is not fully effective. Efforts to offset decreased cerebral perfusion often prove inadequate, resulting in common clinical symptoms such as ischemic stroke and TIAs. Additionally, these fragile collateral networks are prone to bleeding, which can lead to hemorrhagic stroke.

Angiographic studies can provide important insights into the severity of MMD. Nevertheless, there is often a discrepancy between angiographic results and the clinical manifestations of the condition. Some patients may show bilateral occlusion of the internal carotid arteries along with significant collateral vessels but remain asymptomatic. Conversely, individuals with only mild stenosis or near‐occlusive disease, as confirmed by angiography, may experience significant ischemic symptoms. This discrepancy likely reflects the intricate interactions among the various factors that influence cerebral hemodynamics, such as age, systemic condition, the overall quality of the cerebral circulation, and existing collateral networks. The evaluation of cerebral hemodynamics is a valuable surrogate marker for judging the efficacy of compensatory mechanisms^221^ as well as the impending risk of stroke^222^, ^223^ or disease severity.^224^ This is highly advantageous for developing treatment strategies and predicting outcomes in patients with MMD. Over the last 20 years, techniques for assessing changes in cerebral hemodynamics during the perioperative period have also advanced significantly.

### 3.4 Oxygen‐15 positron emission tomography

Oxygen‐15 positron emission tomography (PET) has been extensively used to investigate patients with MMD.^225^, ^226^ PET is the primary functional imaging technique used to assess metabolic processes related to MMD vascular function after bypass surgery. This enables the measurement of various hemodynamic parameters. In addition to the regional CBF (rCBF) and cerebral metabolic rate of oxygen (CMRO2), it can also quantify the regional CBV and regional OEF. Recently, H215O PET has been employed to preoperatively evaluate neuropsychological impairments by measuring territorial cerebrovascular reactivity (CVR), whereas 15O PET has been used to explore cognitive improvement by tracking changes in OEF and CMRO2 after indirect bypass procedures.^227^, ^228^ The primary drawbacks of PET imaging are its limited availability, high cost, and long measurement duration.

### 3.5 Single‑photon emission computed tomography

Single‑photon emission computed tomography (CT) has often been used in research on MMD as well as to assess CBF after surgery, which encompasses both preoperative and postoperative evaluations of cerebral perfusion and vasodilatory capacity.^229^, ^230^, ^231^ It is commonly employed alongside a vasodilatory challenge, such as the acetazolamide test, to evaluate functional reserve. This approach is considerably more accessible. Nevertheless, it lacks exact quantitative precision, and the produced rCBF map needs to be statistically analyzed against a normal control group to identify areas with abnormal perfusion. Additionally, it suffers from low spatial resolution.

### 3.6 Xenon‐enhanced computed tomography

Xenon‐enhanced CT has been a fundamental method for measuring CBF for over 20 years. It has also been extensively used in the study of MMD, and when combined with the acetazolamide challenge, it is often used to assess the appropriateness of revascularization surgery and evaluate postoperative CBF.^232^, ^233^, ^234^, ^235^ The primary disadvantages of this method include a relatively long acquisition time, susceptibility to motion artifacts, and the fact that some patients may find it difficult to tolerate the inhalation of xenon through a face mask during the procedure. Additionally, owing to regulatory constraints, the Xe‐CT CBF technique is not widely accessible in many countries.

### 3.7 Perfusion computed tomography

Perfusion CT is a relatively recent technique used to assess CBF through a first‐pass tracer approach. Its popularity has increased with the increased availability of helical CT scanners that can function in cine mode, and there are several reports of its application in research on MMD.^236^, ^237^, ^238^ Maps of CBF, CBV, and mean transit time can be easily generated. The major benefits of this technique include the widespread global availability of CT scanners, speed of the examination, and minimal significant contraindications. However, drawbacks include radiation exposure and the potential unsuitability of iodinated contrast material for certain patients.

### 3.8 Dynamic susceptibility contrast MRI

Dynamic susceptibility contrast MRI (DSC‐MRI) is currently among the most commonly used MR perfusion imaging methods for quantifying cerebral hemodynamic alterations in the field of neurosurgery. This technique facilitates the identification of suitable candidates for MMD intervention and forecasting of surgical outcomes and associated risks. In research conducted by Ishii et al., the mean transit time assessed using DSC‐MRI revealed minor improvements in arteriogenesis as soon as 2–4 weeks after indirect bypass surgery, which is significantly earlier than that shown when using other imaging techniques.^239^, ^240^ The extent of shortening of the mean transit time is positively associated with the effectiveness of the surgery. Nevertheless, DSC‐MRI has some disadvantages, including the requirement for a contrast agent, limited quantitative reliability, and prolonged resolution times.

### 3.9 Arterial spin labeling MRI

ASL MRI does not require the use of a contrast agent because it uses endogenous water as a tracer—magnetically tagged blood entering the area is used as a natural tracer to evaluate cerebral perfusion at the tissue level.^241^, ^242^ This technique is especially suitable for use in children with MMD.^243^, ^244^, ^245^ Research has shown the clinical importance of ASL‐MRI in assessing hemodynamic dysfunction after surgery.^244^, ^246^ The benefits of this technique include the absence of radiation or need for intravenous contrast, repetition possibility, and the ability to compare CBF values within individual patients and across different examinations. However, a considerable drawback of using this method to investigate MMD is that the assessed CBF values may be lower than the actual values because of extended arterial transit times in stenotic segments and collateral pathways as well as inaccuracies from labeling agents remaining in feeding vessels instead of being delivered to the brain tissue.

### 3.10 Transcranial Doppler ultrasonography

Owing to its noninvasive nature, rapid execution, bedside convenience, and low cost, transcranial Doppler (TCD) is frequently used to track intracranial ischemic conditions. This technique has been employed in numerous studies related to MMD.^247^, ^248^, ^249^ Cerebral vasomotor reactivity assessed using TCD can indicate cerebrovascular reserve, serving as a crucial reference for evaluating hemodynamics prior to surgery.^250^ However, it is highly dependent on the operator and is susceptible to inaccurate results.

### 3.11 Flow probes

All the techniques discussed thus far rely on the indirect assessment of rCBF. However, during revascularization surgery, particularly for MMD, it is possible to directly measure local blood flow. A flexible perivascular flow probe (Charbel, Transonic Systems, Inc.) can be positioned over a target vessel that has been meticulously isolated from the adjacent tissue. This flowmeter utilizes an ultrasonic transit‐time technique to directly measure the volumetric flow in mL/min.

### 3.12 Transcranial color‑coded duplex sonography

Transcranial color‑coded duplex sonography offers real‐time quantitative data on both the donor and recipient arteries, making it an excellent imaging modality for monitoring graft flow after surgery.^251^, ^252^ Quantitative metrics, including peak systolic velocity, end‐diastolic velocity, mean flow volume, pulsatility index, and resistance index, are typically assessed using the trans‐temporal, trans‐orbital, and trans‐foraminal windows.^253^ An imbalance between graft flow and the middle cerebral artery network can lead to postoperative cerebral hyperperfusion.^254^

### 3.13 Blood oxygen level‐dependent functional MRI

Blood oxygen level‐dependent functional MRI (BOLD‐fMRI) is a brain mapping technique that employs deoxyhemoglobin found in blood vessels as an inherent contrast agent to create functional activation maps. This technique is extensively used to track changes in CVR and neurovascular coupling as well as to evaluate the effectiveness of surgery after revascularization.^255^, ^256^ In BOLD‐fMRI studies, the end‐expiratory carbon dioxide concentration has been frequently adjusted to assess CVR using breath‐holding or CO_2_ inhalation methods.^257^, ^258^ Liu et al. have suggested the use of resting‐state BOLD data to assess CVR, thereby eliminating the need for gas inhalation or breath‐holding techniques.^259^, ^260^ BOLD‐fMRI has the potential to become a standard procedure for the pre‐ and postoperative assessment of patients with MMD, particularly in children. Nevertheless, enhancing the stability and repeatability of BOLD imaging remains a significant challenge in the clinical setting.

### 3.14 Intraoperative ultrasound

Intraoperative intravascular ultrasound (IVUS) allows for the accurate placement of a perivascular flow probe over the dissected target vasculature without diminishing the acoustic beam, enabling rapid assessment of graft patency and the correction of any issues with grafts, thereby significantly enhancing the success rate.^261^, ^262^, ^263^ In contrast to indocyanine green video‐angiography, micro‐Doppler imaging not only facilitates the accurate identification of the recipient artery prior to anastomosis, but also allows for the assessment of blood flow velocity and direction after bypass surgery, thereby characterizing hemodynamic conditions.^264^, ^265^

### 3.15 Fluorescence imaging

Indocyanine green and sodium fluorescein are frequently used as imaging agents for fluorescence visualization during surgery. The use of Flow Insight software® (Carl Zeiss, Co.) or Flow 800 software enables the “visualization” of blood flow direction. This type of imaging offers semiquantitative insights into regional hemodynamic changes and can help identify patients at an elevated risk of transient neurological events, enabling adjustments to perioperative management.^266^, ^267^, ^268^ The authors of a previous study have reported that a mismatch ratio between the donor STA and recipient middle cerebral artery as well as insufficient runoff or stagnation of blood flow from the STA may contribute to postoperative cerebral hyperperfusion syndrome in patients with MMD.^269^

A comparable optical imaging method involves the microscopic examination of cortical venous redness, also known as venous reddening. This technique uses digital photography to assess venous oxygen saturation during surgical procedures, providing indirect insights into tissue oxygen metabolism and potential disturbances in CBF. It holds significant value for the immediate detection of cerebral hyperperfusion syndrome following anastomosis.^270^, ^271^

### 3.16 Contrast‑enhanced ultrasound

Contrast‐enhanced ultrasound (CEUS) is an emerging imaging modality that is used to effectively visualize microvascular circulation and cerebral perfusion, providing both qualitative and semiquantitative data.^272^, ^273^ The use of CEUS to assess revascularization in patients with MMD is not widely recognized among neurosurgeons. The authors of a recent investigation using a rat model of middle cerebral artery occlusion reported that transcranial CEUS revealed significant reductions in blood volume, flow velocity, and cerebral perfusion in both the cortex and striatum during ischemic stroke. Following vascular recanalization, both blood volume and perfusion were elevated to twice the baseline level, indicating acute cerebral hyperperfusion syndrome.^274^ This study simulated the surgical bypass mechanism for MMD and established a basis for future intraoperative CEUS assessments in patients with MMD.

## 4 THERAPEUTIC INTERVENTIONS

MMD treatment includes medical, endovascular, and surgical treatments. Of these, surgical treatment is currently recognized as the most effective (Table 2).

**TABLE 2:** Clinical research into moyamoya disease (MMD).

Authors	Year of publication	Country of publication	Types of clinical studies	Number of patients/controls	Findings
Pang et al.^275^	2021	Korea	Retrospective cohort study	243	Antiplatelet drugs improve ischemic symptoms in adult patients with MMD.
Yamada et al.^276^	2015	Japan	Prospective cohort study	735/541	Revascularization surgery may suppress recurrent ischemic attacks in patients with MMD.
Zhao et al.^277^	2017	China	Retrospective cohort study	59/138	Postoperative aspirin does not affect the incidence of ischemic or bleeding events in adult patients with MMD undergoing revascularization surgery, but may improve short‐term outcomes.
Seo et al.^278^	2021	Korea	Retrospective cohort study	25,978	Antiplatelet therapy is associated with substantial improvement in survival in patients with MMD.
Onozuka et al.^279^	2016	Japan	Retrospective cohort study	1925	Prehospital antiplatelet use should be considered when evaluating outcomes of patients with nonhemorrhagic MMD.
Chiba et al.^280^	2018	Japan	Prospective cohort study	68	Cilostazol improves cerebral perfusion better than clopidogrel does in adult patients with symptomatically ischemic MMD not accompanied by misery perfusion.
Aihara et al.^281^	2021	Japan	Retrospective cohort study	35	Aspirin is capable of alleviating headache symptoms in children with MMD.
Yeon et al.^282^	2010	Korea	Retrospective cohort study	249	The probability of large aneurysms occurring in patients with ischemic MMD increases with age.
Kawaguchi et al.^283^	1996	Japan	Retrospective cohort study	111	It is recommended to perform surgical intervention on aneurysms around the circle of Willis in MMD. It is not advised to operate directly on aneurysms on the basal ganglia or collateral vessels in MMD.
Yu et al.^284^	2010	China	Retrospective cohort study	13	Intracranial aneurysms in patients with MMD can have favorable prognoses through endovascular treatment.
Cho et al.^285^	2014	Korea	Retrospective cohort study	60	Combined revascularization surgery resulted in ameliorating symptoms and preventing the recurrence of stroke.
Miyamoto et al.^286^	2014	Japan	Multicentered, prospective, randomized, controlled trial study	80	The preventive effect of direct bypass resulted in less rebleeding.
Karasawa et al.^287^	1978	Japan	Retrospective cohort study	23	The superficial temporal artery (STA)–MCA anastomosis appears to be an efficacious approach for the treatment of MMD.
Nielsen et al.^288^	2021 2020	United States	Retrospective cohort study	138	Adult patients with MMD are better suited for direct bypass surgery.
Houkin et al.^289^	2000	Japan	Retrospective cohort study	85	Direct bypass surgery is effective for adult patients with MMD.
Czabanka et al.^290^	2011	Germany	Prospective cohort study	30	For adult patients with MMD, combined revascularization surgery is superior to single surgery.
Deng et al.^291^	2017	China	Retrospective cohort study	220	In preventing recurrent strokes in adult patients with ischemic MMD, direct bypass surgery is more effective than is indirect bypass surgery.
Ding et al.^292^	2019	China	Prospective cohort study	30	Remote ischemic conditioning can alleviate the ischemic symptoms in patients with MMD.

Abbreviations: MCA, middle cerebral artery.

### 4.1 Medical treatment

Long‐term administration of antiplatelet agents is effective in mitigating TIA in patients with MMD.^275^ However, current evidence suggests that the use of antiplatelet agents does not reduce the incidence of ischemic stroke in this patient population,^275^, ^276^ while some studies have found that antiplatelet agents reduce or do not increase the incidence of bleeding,^275^, ^276^, ^277^ or even improve long‐term prognosis.^278^, ^279^ Cilostazol has been associated with improved cerebral perfusion and lower mortality rates.^278^, ^280^ Indications for the treatment of asymptomatic MMD are currently being evaluated in the AMORE trial.^293^ In patients undergoing direct or combined revascularization, although aspirin does not reduce the rate of postoperative ischemic stroke or increase graft patency, it also does not increase the risk of hemorrhage.^277^ In addition, aspirin plays a role in the management of headache in children.^281^

### 4.2 Endovascular treatment

There is no evidence that endovascular treatment improves the natural history of MMD, and the limited available data do not support this treatment method given the risk of complications.^294^

Managing aneurysms associated with MMD presents considerable challenges to neurosurgeons, primarily because of the intricate nature of endovascular embolization and clipping procedures. The incidence of intracranial aneurysms in patients with MMD ranges from 3.4% to 14.8%, which is significantly higher than the estimated incidence of 1%–3% in the general population.^282^, ^283^ Aneurysm rupture can cause intracranial hemorrhage, and surgical treatment of ruptured aneurysms is recommended in the literature.^295^, ^296^ However, the outcomes of aneurysm clipping or endovascular embolization are generally unsatisfactory, likely because of interference with the existing collateral circulation.^284^ For ruptured anterior circulation aneurysms, clipping or embolization should be performed to prevent re‐rupture. However, controversy remains regarding unruptured anterior circulation aneurysms, which may spontaneously disappear with progressive occlusion of the terminal internal carotid, anterior cerebral, and middle cerebral arteries.^297^, ^298^ In cases of posterior circulation aneurysms, craniotomy clipping is challenging and risky, making endovascular embolization the preferred treatment approach.

### 4.3 Revascularization surgery

The main objective of surgical intervention for MMD is to enhance blood circulation in regions with reduced perfusion, typically indicated by recurring symptoms of cerebral ischemia and/or diminished perfusion observed on imaging studies.^299^ At present, the accepted idea behind the treatment of MMD is to improve blood supply to the ischemic area by increasing CBF rather than reversing the pathogenesis of MMD. Revascularization is thought to be useful for alleviating ischemic injury. However, whether hemorrhagic or ischemic MMD is present, revascularization surgery can only reduce but not completely prevent the occurrence of stroke. In a study involving 77 cerebral hemispheres, the annual rates of symptomatic hemorrhage and cerebral infarction in the surgical hemisphere were 0.4% and 0.2%, respectively.^285^ The hemorrhagic MMD type is associated with a higher likelihood of rebleeding and a worse prognosis than the ischemic type is.^300^

STA–middle cerebral artery anastomosis has been used to treat MMD in children and adults since the 1970s^287^ and is the most common surgery for direct revascularization. Other direct revascularization procedures such as STA–anterior cerebral artery, STA–posterior cerebral artery, and occipital artery–PCA anastomoses have been used in different areas of bypass. In adults with MMD, direct or combined revascularization is more widely used and more effective in preventing ischemic stroke.^288^ However, direct revascularization is more challenging in children because the bypass patency rate is lower than it is in adults (53% vs. 94%).^289^

Indirect revascularization was introduced simultaneously with direct revascularization in the 1970s. The most typical type of indirect revascularization is EMS. To date, various indirect revascularization methods have been developed, including encephalo‐arterio‐synangiosis, encephalo‐myo‐arterio‐synangiosis, encephalo‐duo‐synangiosis, encephalo‐duro‐arterio‐synangiosis, encephalo‐duroarterio‐myo‐synangiosis, encephalo‐duro‐galeo (periosteal)‐synangiosis, and various combinations of these. Nevertheless, there is insufficient evidence to suggest that indirect revascularization is more effective than is direct revascularization. Indirect revascularization depends on neovascularization of the cortical surface, and the angiogenic mechanism of the pedicled graft dictates that neovascularization usually takes several months to develop. Indirect revascularization is easier to implement, but vessel growth is unpredictable. In children with MMD, indirect revascularization generally provides satisfactory stroke prevention.^289^, ^290^

However, a meta‐analysis indicated that combined bypass surgery outperformed both direct and indirect bypass procedures when performed individually, resulting in better long‐term outcomes in terms of clinical, angiographic, and hemodynamic conditions.^301^ Direct or combined revascularization generally results in better vessel growth than does indirect revascularization. Another meta‐analysis showed that direct revascularization is more effective than is indirect revascularization in reducing the rate of further stroke.^302^ Meanwhile, another cohort study with long‐term follow‐up of patients with MMD revealed that either direct or combined revascularization is more effective than is indirect revascularization for the prevention of ischemic stroke.^291^ This difference may be due to the fact that vascular plasticity is closely associated with age and growth.^290^

The only randomized, controlled clinical trial that has been conducted to date has shown a reduction in the incidence of rebleeding after direct bilateral revascularization in hemorrhagic MMD, which has a preventive effect on rebleeding.^300^ There is no consensus on the need for and timing of surgical intervention.

### 4.4 Other treatment

Some researchers have attempted to use remote ischemic conditioning devices for stroke prevention in patients with ischemic MMD.^292^ They discovered that remote ischemic conditioning can positively impact the management of ischemic events induced by MMD, alleviate symptoms, and enhance cerebral perfusion. However, owing to the small sample size, the effect is uncertain.

## 5 CONCLUSIONS

According to current research, MMD may not be a hereditary disorder caused by a single gene. Multiple genes—including those encoding inflammatory, immune‐related, and environmental factors—may initiate or contribute to the pathogenesis of MMD. Therefore, research on MMD cannot be limited to the simple models used in the past. Traditional studies that focus on a single variable may not capture the complex multifactorial nature of MMD. Although our understanding of MMD has become more precise and offers improved diagnostic and treatment options, several limitations remain:

- lack of successful animal models
- difficulties in obtaining pathological cerebral vascular specimens
- lack of convenient screening measures for asymptomatic patients with MMD
- lack of effective endovascular therapy

Revascularization surgery continues to be the primary treatment for MMD, as no effective drugs have been developed to prevent or slow the progression of the disease. Therefore, early detection, accurate diagnosis, the identification of biological markers, and the discovery of new therapeutic targets are important directions for future research. At present, many researchers believe that MMD is not simply caused by a single gene mutation but also involves immune regulation, epigenetics, actin remodeling, metabolic abnormalities, and other factors. Multiomic research at both the central and peripheral levels can provide a more comprehensive picture of the pathological mechanisms of MMD. The following are potential directions for future research on MMD:

- advancements in multifactorial pathogenesis mechanisms
- organoids for MMD
- quantifiable diagnostic indicators
- effective drugs for new therapeutic targets

After reviewing and discussing the literature on MMD over several decades, we conclude that the scope of MMD research should be broadened. Genetics may be an important but not exclusive factor in MMD. In the absence of animal models, organoid MMD models may provide a meaningful foundation for future research on disease mechanisms and drug screening. Therefore, further studies should focus on the development of in vivo and in vitro models to better understand the complexity of MMD.

## AUTHOR CONTRIBUTIONS

Shihao He, Yuanli Zhao, and Gary K. Steinberg conceived and designed the study. Shihao He, Michelle Y. Cheng, Zhenyu Zhou, Xiaokuan Hao, Terrance Chiang, Yanru Wang, Junze Zhang, Xilong Wang, Xun Ye, and Rong Wang searched the literature. Shihao He, Michelle Y. Cheng, and Zhenyu Zhou wrote the manuscript. All the authors have read and approved the final manuscript.

## CONFLICT OF INTEREST STATEMENT

The authors declare no conflicts of interest.

## ETHICS STATEMENT

Not applicable.

## Data Availability

Not applicable.

## References

[1] Scott R , Smith ER . Moyamoya disease and moyamoya syndrome. N Engl J Med. 2009;360(12):1226‐1237. 19297575 10.1056/NEJMra0804622

[2] Sato Y , Kazumata K , Nakatani E , Houkin K , Kanatani Y . Characteristics of moyamoya disease based on national registry data in Japan. Stroke. 2019;50(8):1973‐1980. 31234758 10.1161/STROKEAHA.119.024689

[3] Kuriyama S , Kusaka Y , Fujimura M , et al. Prevalence and clinicoepidemiological features of moyamoya disease in Japan. Stroke. 2008;39(1):42‐47. 18048855 10.1161/STROKEAHA.107.490714

[4] Wakai K , Tamakoshi A , Ikezaki K , et al. Epidemiological features of moyamoya disease in Japan: findings from a nationwide survey. Clin Neurol Neurosurg. 1997;99(2):S1‐S5. 10.1016/s0303-8467(97)00031-0 9409395

[5] Bao X‐Y , Wang Q‐N , Zhang Y , et al. Epidemiology of moyamoya disease in China: single‐center, population‐based study. World Neurosurg. 2019;122:e917‐e923. 30404059 10.1016/j.wneu.2018.10.175

[6] Starke RM , Crowley RW , Maltenfort M , et al. Moyamoya disorder in the United States. Neurosurgery. 2012;71(1):93‐99. 22418580 10.1227/NEU.0b013e318253ab8e

[7] Kainth D , Chaudhry SA , Kainth H , Suri FK , Qureshi AI . Epidemiological and clinical features of moyamoya disease in the USA. Neuroepidemiology. 2013;40(4):282‐287. 23445954 10.1159/000345957

[8] Ihara M , Yamamoto Y , Hattori Y , et al. Moyamoya disease: diagnosis and interventions. Lancet Neurol. 2022;21(8):747‐758. 35605621 10.1016/S1474-4422(22)00165-X

[9] Gonzalez NR , Amin‐Hanjani S , Bang OY , et al. Adult moyamoya disease and syndrome: current perspectives and future directions: a scientific statement from the American Heart Association/American Stroke Association. Stroke. 2023;54(10):e465‐e479. 37609846 10.1161/STR.0000000000000443

[10] Tokairin K , Hamauchi S , Ito M , et al. Vascular smooth muscle cell derived from IPS cell of moyamoya disease—comparative characterization with endothelial cell transcriptome. J Stroke Cerebrovasc Dis. 2020;29(12):105305. 32992193 10.1016/j.jstrokecerebrovasdis.2020.105305

[11] Blecharz KG , Frey D , Schenkel T , et al. Autocrine release of angiopoietin‐2 mediates cerebrovascular disintegration in moyamoya disease. J Cereb Blood Flow Metab. 2017;37(4):1527‐1539. 27381827 10.1177/0271678X16658301 PMC5453470

[12] Kuroda S , Fujimura M , Takahashi J , et al. Diagnostic criteria for moyamoya disease ‐ 2021 revised version. Neurol Med Chir (Tokyo). 2022;62(7):307‐312. 35613882 10.2176/jns-nmc.2022-0072 PMC9357455

[13] Fujimura M , Tominaga T , Kuroda S , et al. 2021 Japanese guidelines for the management of moyamoya disease: guidelines from the research committee on moyamoya disease and Japan Stroke Society. Neurol Med Chir. 2022;62(4):165‐170. 10.2176/jns-nmc.2021-0382 PMC9093674 35197402

[14] Research Committee on the Pathology and Treatment of Spontaneous Occlusion of the Circle of Willis; Health Labour Sciences Research Grant for Research on Measures for Infractable Diseases . Guidelines for diagnosis and treatment of moyamoya disease (spontaneous occlusion of the circle of Willis). Neurol Med Chir. 2012;52(5):245‐266. 10.2176/nmc.52.245 22870528

[15] Mukerji N , Cook DJ , Steinberg GK . Is local hypoperfusion the reason for transient neurological deficits after STA‐MCA bypass for moyamoya disease?. J Neurosurg. 2015;122(1):90‐94. 25343178 10.3171/2014.8.JNS132413

[16] Fujimura M , Mugikura S , Kaneta T , Shimizu H , Tominaga T . Incidence and risk factors for symptomatic cerebral hyperperfusion after superficial temporal artery‐middle cerebral artery anastomosis in patients with moyamoya disease. Surg Neurol. 2009;71(4):442‐447. 18514264 10.1016/j.surneu.2008.02.031

[17] Kaku Y , Iihara K , Nakajima N , et al. Cerebral blood flow and metabolism of hyperperfusion after cerebral revascularization in patients with moyamoya disease. J Cereb Blood Flow Metab. 2012;32(11):2066‐2075. 22850406 10.1038/jcbfm.2012.110 PMC3493997

[18] Lee SU , Oh CW , Kwon OK , et al. Surgical treatment of adult moyamoya disease. Curr Treat Options Neurol. 2018;20(7):20. 29808372 10.1007/s11940-018-0511-8

[19] Takekawa Y , Umezawa T , Ueno Y , Sawada T , Kobayashi M . Pathological and immunohistochemical findings of an autopsy case of adult moyamoya disease. Neuropathology. 2004;24(3):236‐242. JNojotJSoN. 15484702 10.1111/j.1440-1789.2004.00550.x

[20] Takagi Y , Kikuta K , Nozaki K . Hashimoto NJNm‐c. Histological features of middle cerebral arteries from patients treated for moyamoya disease. Neurol Med Chir (Tokyo). 2007;47(1):1‐4. 17245006 10.2176/nmc.47.1

[21] Kuroda S , Houkin K . Moyamoya disease: current concepts and future perspectives. Lancet Neurol. 2008;7(11):1056‐1066. 18940695 10.1016/S1474-4422(08)70240-0

[22] Takagi Y , Hermanto Y , Takahashi JC , et al. Histopathological characteristics of distal middle cerebral artery in adult and pediatric patients with moyamoya disease. Neurol Med Chir (Tokyo). 2016;56(6):345‐349. 27087193 10.2176/nmc.oa.2016-0031 PMC4908078

[23] Takagi Y , Kikuta K , Sadamasa N , Nozaki K , Hashimoto NJN . Caspase‐3‐dependent apoptosis in middle cerebral arteries in patients with moyamoya disease. Neurosurgery. 2006;59(4):894‐900. discussion 900–1. 17038954 10.1227/01.NEU.0000232771.80339.15

[24] Lim M , Cheshier S , Steinberg GK . New vessel formation in the central nervous system during tumor growth, vascular malformations, and moyamoya. Curr Neurovasc Res. 2006;3(3):237‐245. 16918387 10.2174/156720206778018730

[25] Oka K , Yamashita M , Sadoshima S , Tanaka K . Pathological anatomy, histology. Cerebral haemorrhage in moyamoya disease at autopsy. Virchows Arch A Pathol Anat Histol. 1981;392(3):247‐261. 7269227 10.1007/BF02155663

[26] Yamashita M , Tanaka K , Matsuo T , Yokoyama K , Fujii T , Sakamoto H . Cerebral dissecting aneurysms in patients with moyamoya disease. Report of two cases. J Neurosurg. 1983;58(1):120‐125. 6847898 10.3171/jns.1983.58.1.0120

[27] Li B , Wang C‐C , Zhao Z‐Z , et al. A histological, ultrastructural and immunohistochemical study of superficial temporal arteries and middle meningeal arteries in moyamoya disease. Acta Pathol Japon. 1991;41(7):521‐530. 10.1111/j.1440-1827.1991.tb02517.x 1755318

[28] Dei Cas M , Carrozzini T , Pollaci G , et al. Plasma lipid profiling contributes to untangle the complexity of moyamoya arteriopathy. Int J Mol Sci. 2021;22(24):13410. 34948203 10.3390/ijms222413410 PMC8708587

[29] Lu J , Wang J , Lin Z , et al. MMP‐9 as a biomarker for predicting hemorrhagic strokes in moyamoya disease. Front Neurol. 2021;12:721118. 34531816 10.3389/fneur.2021.721118 PMC8438170

[30] He S , Zhang J , Liu Z , et al. Upregulated cytoskeletal proteins promote pathological angiogenesis in moyamoya disease. Stroke. 2023;54(12):3153‐3164. 37886851 10.1161/STROKEAHA.123.044476

[31] Wang Z , Ji C , Han Q , Wang Z , Huang Y . Data‐independent acquisition‐based serum proteomic profiling of adult moyamoya disease patients reveals the potential pathogenesis of vascular changes. J Mol Neurosci. 2022;72(12):2473‐2485. 36520382 10.1007/s12031-022-02092-w

[32] Kim S , Yoo J , Cho B , et al. Elevation of CRABP‐I in the cerebrospinal fluid of patients with moyamoya disease. Stroke. 2003;34(12):2835‐2841. 14605320 10.1161/01.STR.0000100159.43123.D7

[33] Maruwaka M , Yoshikawa K , Okamoto S , et al. Biomarker research for moyamoya disease in cerebrospinal fluid using surface‐enhanced laser desorption/ionization time‐of‐flight mass spectrometry. J Stroke Cerebrovasc Dis. 2015;24(1):104‐111. 25440344 10.1016/j.jstrokecerebrovasdis.2014.07.028

[34] He S , Liang J , Xue G , et al. RNA profiling of sEV (small extracellular vesicles)/exosomes reveals biomarkers and vascular endothelial dysplasia with moyamoya disease. J Cereb Blood Flow Metab. 2023;43(7):1194‐1205. 36883376 10.1177/0271678X231162184 PMC10291455

[35] Wang X , Han C , Jia Y , Wang J , Ge W , Duan L . Proteomic profiling of exosomes from hemorrhagic moyamoya disease and dysfunction of mitochondria in endothelial cells. Stroke. 2021;52(10):3351‐3361. 34334053 10.1161/STROKEAHA.120.032297

[36] Jeon JP , Yun T , Jin X , et al. 1H‐NMR‐based metabolomic analysis of cerebrospinal fluid from adult bilateral moyamoya disease: comparison with unilateral moyamoya disease and atherosclerotic stenosis. Medicine (Baltimore). 2015;94(17):e629. 25929894 10.1097/MD.0000000000000629 PMC4603033

[37] Geng C , Cui C , Guo Y , et al. Metabolomic profiling revealed potential biomarkers in patients with moyamoya disease. Front Neurosci. 2020;14:308. 32372905 10.3389/fnins.2020.00308 PMC7186471

[38] He S , Wang Y , Liu Z , et al. Metabolomic signatures associated with pathological angiogenesis in moyamoya disease. Clin Transl Med. 2023;13(12):e1492. 38037492 10.1002/ctm2.1492 PMC10689969

[39] Liu X , Jin F , Wang C , et al. Targeted metabolomics analysis of serum amino acid profiles in patients with moyamoya disease. Amino Acids. 2022;54(1):137‐146. 34800175 10.1007/s00726-021-03100-w

[40] Liu W , Morito D , Takashima S , et al. Identification of RNF213 as a susceptibility gene for moyamoya disease and its possible role in vascular development. PLoS One. 2011;6(7):e22542. 21799892 10.1371/journal.pone.0022542 PMC3140517

[41] Hitomi T , Habu T , Kobayashi H , et al. Downregulation of securin by the variant RNF213 R4810K (rs112735431, G>A) reduces angiogenic activity of induced pluripotent stem cell‐derived vascular endothelial cells from moyamoya patients. Biochem Biophys Res Commun. 2013;438(1):13‐19. 23850618 10.1016/j.bbrc.2013.07.004

[42] Hitomi T , Habu T , Kobayashi H , et al. The moyamoya disease susceptibility variant RNF213 R4810K (rs112735431) induces genomic instability by mitotic abnormality. Biochem Biophys Res Commun. 2013;439(4):419‐426. 23994138 10.1016/j.bbrc.2013.08.067

[43] Kobayashi H , Matsuda Y , Hitomi T , et al. Biochemical and functional characterization of RNF213 (Mysterin) R4810K, a susceptibility mutation of moyamoya disease, in angiogenesis in vitro and in vivo. J Am Heart Assoc. 2015;4(7):e002146. 26126547 10.1161/JAHA.115.002146 PMC4608092

[44] Pollaci G , Gorla G , Potenza A , et al. Novel multifaceted roles for RNF213 protein. Int J Mol Sci. 2022;23(9):4492. 35562882 10.3390/ijms23094492 PMC9099590

[45] Ohkubo K , Sakai Y , Inoue H , et al. Moyamoya disease susceptibility gene RNF213 links inflammatory and angiogenic signals in endothelial cells. Sci Rep. 2015;5:13191. 26278786 10.1038/srep13191 PMC4538604

[46] Apte RS , Chen DS , Ferrara N . VEGF in signaling and disease: beyond discovery and development. Cell. 2019;176(6):1248‐1264. 30849371 10.1016/j.cell.2019.01.021 PMC6410740

[47] Sakamoto S , Kiura Y , Yamasaki F , et al. Expression of vascular endothelial growth factor in dura mater of patients with moyamoya disease. Neurosurg Rev. 2008;31(1):77‐81. discussion 81. 17912564 10.1007/s10143-007-0102-8

[48] Park YS , Jeon YJ , Kim HS , et al. The role of VEGF and KDR polymorphisms in moyamoya disease and collateral revascularization. PLoS One. 2012;7(10):e47158. 23077562 10.1371/journal.pone.0047158 PMC3470587

[49] Kang HS , Kim JH , Phi JH , et al. Plasma matrix metalloproteinases, cytokines and angiogenic factors in moyamoya disease. J Neurol Neurosurg Psychiatry. 2010;81(6):673‐678. 19965844 10.1136/jnnp.2009.191817

[50] Marushima A , Nieminen M , Kremenetskaia I , et al. Balanced single‐vector co‐delivery of VEGF/PDGF‐BB improves functional collateralization in chronic cerebral ischemia. J Cereb Blood Flow Metab. 2020;40(2):404‐419. 30621518 10.1177/0271678X18818298 PMC7370608

[51] Hiramatsu M , Hishikawa T , Tokunaga K , et al. Combined gene therapy with vascular endothelial growth factor plus apelin in a chronic cerebral hypoperfusion model in rats. J Neurosurg. 2017;127(3):679‐686. 28009234 10.3171/2016.8.JNS16366

[52] Wu MY , Hill CS . TGF‐beta superfamily signaling in embryonic development and homeostasis. Dev Cell. 2009;16(3):329‐343. 19289080 10.1016/j.devcel.2009.02.012

[53] Hojo M , Hoshimaru M , Miyamoto S , et al. Role of transforming growth factor‐beta1 in the pathogenesis of moyamoya disease. J Neurosurg. 1998;89(4):623‐629. 9761057 10.3171/jns.1998.89.4.0623

[54] Chen Y , Tang M , Li H , Liu H , Wang J , Huang J . TGFbeta1 as a predictive biomarker for collateral formation within ischemic moyamoya disease. Front Neurol. 2022;13:899470. 35873760 10.3389/fneur.2022.899470 PMC9301205

[55] Wang C , Sun C , Zhao Y , et al. RNF213 gene silencing upregulates transforming growth factor beta1 expression in bone marrow‐derived mesenchymal stem cells and is involved in the onset of moyamoya disease. Exp Ther Med. 2021;22(3):1024. 34373710 10.3892/etm.2021.10456 PMC8343649

[56] Yamamoto M , Aoyagi M , Tajima S , et al. Increase in elastin gene expression and protein synthesis in arterial smooth muscle cells derived from patients with moyamoya disease. Stroke. 1997;28(9):1733‐1738. 9303017 10.1161/01.str.28.9.1733

[57] Hellström M , Gerhardt H , Kalén M , et al. Lack of pericytes leads to endothelial hyperplasia and abnormal vascular morphogenesis. J Cell Biol. 2001;153(3):543‐553. 11331305 10.1083/jcb.153.3.543 PMC2190573

[58] Aoyagi M , Fukai N , Matsushima Y , Yamamoto M , Yamamoto K . Kinetics of 125I‐PDGF binding and down‐regulation of PDGF receptor in arterial smooth muscle cells derived from patients with moyamoya disease. J Cell Physiol. 1993;154(2):281‐288. 8425908 10.1002/jcp.1041540210

[59] Yamamoto M , Aoyagi M , Fukai N , Matsushima Y , Yamamoto KJS . Differences in cellular responses to mitogens in arterial smooth muscle cells derived from patients with moyamoya disease. Stroke. 1998;29(6):1188‐1193. 9626293 10.1161/01.str.29.6.1188

[60] Hayashi T , Yamamoto S , Hamashima T , Mori H , Sasahara M , Kuroda S . Critical role of platelet‐derived growth factor‐alpha in angiogenesis after indirect bypass in a murine moyamoya disease model. J Neurosurg. 2020;134(5):1535‐1543. 32442967 10.3171/2020.3.JNS193273

[61] Wang LS , Wang H , Zhang QL , Yang ZJ , Kong FX , Wu CT . Hepatocyte growth factor gene therapy for ischemic diseases. Hum Gene Ther. 2018;29(4):413‐423. 29409352 10.1089/hum.2017.217

[62] Nanba R , Kuroda S , Ishikawa T , Houkin K , Iwasaki YJS . Increased expression of hepatocyte growth factor in cerebrospinal fluid and intracranial artery in moyamoya disease. Stroke. 2004;35(12):2837‐2842. 15528455 10.1161/01.STR.0000148237.13659.e6

[63] Abhinav K , Lee AG , Pendharkar AV , et al. Comprehensive profiling of secreted factors in the cerebrospinal fluid of moyamoya disease patients. Transl Stroke Res. 2023;15(2):399‐408. 36745304 10.1007/s12975-023-01135-7 PMC10891229

[64] Reuss B , von Bohlen und Halbach O . Fibroblast growth factors and their receptors in the central nervous system. Cell Tissue Res. 2003;313(2):139‐157. 12845521 10.1007/s00441-003-0756-7

[65] Malek A , Connors S , Robertson R , Folkman J , Scott R . Elevation of cerebrospinal fluid levels of basic fibroblast growth factor in moyamoya and central nervous system disorders. Pediatr Neurosurg. 1997;27(4):182‐189. 9577971 10.1159/000121249

[66] Bedini G , Blecharz K , Nava S , et al. Vasculogenic and angiogenic pathways in moyamoya disease. Curr Med Chem. 2016;23(4):315‐345. 26861126 10.2174/092986732304160204181543

[67] Mertens R , Graupera M , Gerhardt H , et al. The genetic basis of moyamoya disease. Transl Stroke Res. 2022;13(1):25‐45. 34529262 10.1007/s12975-021-00940-2 PMC8766392

[68] Baba T , Houkin K , Kuroda S . Novel epidemiological features of moyamoya disease. J Neurol Neurosurg Psychiatry. 2008;79(8):900‐904. 18077479 10.1136/jnnp.2007.130666

[69] Mineharu Y , Takenaka K , Yamakawa H , et al. Inheritance pattern of familial moyamoya disease: autosomal dominant mode and genomic imprinting. J Neurol Neurosurg Psychiatry. 2006;77(9):1025‐1029. 16788009 10.1136/jnnp.2006.096040 PMC2077755

[70] Nanba R , Kuroda S , Tada M , Ishikawa T , Houkin K , Iwasaki Y . Clinical features of familial moyamoya disease. Child's Nerv Syst: ChNS. 2006;22(3):258‐262. 16195874 10.1007/s00381-005-1230-5

[71] Kang HS , Kim SK , Cho BK , Kim YY , Hwang YS , Wang KC . Single nucleotide polymorphisms of tissue inhibitor of metalloproteinase genes in familial moyamoya disease. Neurosurgery. 2006;58(6):1074‐1080. discussion 1074–80. 16723886 10.1227/01.NEU.0000215854.66011.4F

[72] Paez MT , Yamamoto T . Single nucleotide polymorphisms of tissue inhibitor of metalloproteinase genes in familial moyamoya disease. Neurosurgery. 2007;60(3):E582. Author reply E582. 10.1227/01.NEU.0000255365.25066.CD 17327779

[73] Fujimura M , Watanabe M , Narisawa A , Shimizu H , Tominaga T . Increased expression of serum matrix metalloproteinase‐9 in patients with moyamoya disease. Surg Neurol. 2009;72(5):476‐480. discussion 480. 19147196 10.1016/j.surneu.2008.10.009

[74] Li H , Zhang ZS , Liu W , et al. Association of a functional polymorphism in the MMP‐3 gene with moyamoya disease in the Chinese Han population. Cerebrovasc Dis (Basel, Switzerland). 2010;30(6):618‐625. 10.1159/000319893 20948207

[75] Roder C , Peters V , Kasuya H , et al. Common genetic polymorphisms in moyamoya and atherosclerotic disease in Europeans. Child's Nerv Syst: ChNS. 2011;27(2):245‐252. 20694560 10.1007/s00381-010-1241-8

[76] Wang X , Zhang Z , Liu W , et al. Impacts and interactions of PDGFRB, MMP‐3, TIMP‐2, and RNF213 polymorphisms on the risk of moyamoya disease in Han Chinese human subjects. Gene. 2013;526(2):437‐442. 23769926 10.1016/j.gene.2013.05.083

[77] Ma J , You C . Association between matrix metalloproteinase‐3 gene polymorphism and moyamoya disease. J Clin Neurosci. 2015;22(3):479‐482. 25564266 10.1016/j.jocn.2014.08.034

[78] Wang X , Wang Y , Nie F , et al. Association of genetic variants with moyamoya disease in 13 000 individuals: a meta‐analysis. Stroke. 2020;51(6):1647‐1655. 32390555 10.1161/STROKEAHA.120.029527

[79] Guo DC , Papke CL , Tran‐Fadulu V , et al. Mutations in smooth muscle alpha‐actin (ACTA2) cause coronary artery disease, stroke, and moyamoya disease, along with thoracic aortic disease. Am J Hum Genet. 2009;84(5):617‐627. 19409525 10.1016/j.ajhg.2009.04.007 PMC2680995

[80] Roder C , Peters V , Kasuya H , et al. Analysis of ACTA2 in European moyamoya disease patients. Eur J Paediatr Neurol: EJPN. 2011;15(2):117‐122. 20970362 10.1016/j.ejpn.2010.09.002

[81] Shimojima K , Yamamoto T . ACTA2 is not a major disease‐causing gene for moyamoya disease. J Hum Genet. 2009;54(11):687‐688. 19745835 10.1038/jhg.2009.91

[82] Hu FY , Zheng HB , Xu YM , Jiang Y , Zhou D . ACTA2 is not a major genetic risk gene for Chinese patients with moyamoya disease. Int J Stroke. 2013;8(7):E43. 24024919 10.1111/ijs.12074

[83] Hojo M , Hoshimaru M , Miyamoto S , et al. Role of transforming growth factor‐beta1 in the pathogenesis of moyamoya disease. J Neurosurg. 1998;89(4):623‐629. 9761057 10.3171/jns.1998.89.4.0623

[84] Roder C , Peters V , Kasuya H , et al. Polymorphisms in TGFB1 and PDGFRB are associated with moyamoya disease in European patients. Acta Neurochir. 2010;152(12):2153‐2160. 20571834 10.1007/s00701-010-0711-9

[85] Liu C , Roder C , Schulte C , et al. Analysis of TGFB1 in European and Japanese moyamoya disease patients. Eur J Med Genet. 2012;55(10):531‐534. 22659181 10.1016/j.ejmg.2012.05.002

[86] He J , Wang R , Zhang D , Zhang Y , Zhang Q , Zhao J . Expression of circulating vascular endothelial growth factor‐antagonizing cytokines and vascular stabilizing factors prior to and following bypass surgery in patients with moyamoya disease. Exp Ther Med. 2014;8(1):302‐308. 24944638 10.3892/etm.2014.1713 PMC4061224

[87] Kitahara T , Okumura K , Semba A , Yamaura A , Makino H . Genetic and immunologic analysis on moya‐moya. J Neurol Neurosurg Psychiatry. 1982;45(11):1048‐1052. 6983566 10.1136/jnnp.45.11.1048 PMC491644

[88] Aoyagi M , Ogami K , Matsushima Y , Shikata M , Yamamoto M , Yamamoto K . Human leukocyte antigen in patients with moyamoya disease. Stroke. 1995;26(3):415‐417. 7886716 10.1161/01.str.26.3.415

[89] Inoue TK , Ikezaki K , Sasazuki T , Matsushima T , Fukui M . Analysis of class II genes of human leukocyte antigen in patients with moyamoya disease. Clin Neurol Neurosurg. 1997;99(suppl 2):S234‐S237. 9409445 10.1016/s0303-8467(97)00051-6

[90] Han H , Pyo CW , Yoo DS , Huh PW , Cho KS , Kim DS . Associations of moyamoya patients with HLA class I and class II alleles in the Korean population. J Korean Med Sci. 2003;18(6):876‐880. 14676447 10.3346/jkms.2003.18.6.876 PMC3055136

[91] Hong SH , Wang KC , Kim SK , Cho BK , Park MH . Association of HLA‐DR and ‐DQ genes with familial moyamoya disease in Koreans. J Korean Neurosurg Soc. 2009;46(6):558‐563. 20062572 10.3340/jkns.2009.46.6.558 PMC2803272

[92] Kraemer M , Horn PA , Roder C , et al. Analysis of human leucocyte antigen genes in Caucasian patients with idiopathic moyamoya angiopathy. Acta Neurochir. 2012;154(3):445‐454. 22234791 10.1007/s00701-011-1261-5

[93] Tashiro R , Niizuma K , Khor SS , et al. Identification of HLA‐DRB1*04:10 allele as risk allele for Japanese moyamoya disease and its association with autoimmune thyroid disease: a case‐control study. PLoS One. 2019;14(8):e0220858. 31412073 10.1371/journal.pone.0220858 PMC6693760

[94] Wan J , Ling W , Zhengshan Z , Xianbo Z , Lian D , Kai W . Association of HLA‐DQA2 and HLA‐B with moyamoya disease in the Chinese Han population. Neurol Genet. 2021;7(3):e592. 34095496 10.1212/NXG.0000000000000592 PMC8176556

[95] Park YS , Min KT , Kim TG , et al. Age‐specific eNOS polymorphisms in moyamoya disease. Child's Nerv Syst: ChNS. 2011;27(11):1919‐1926. 21691823 10.1007/s00381-011-1504-z

[96] Duan L , Wei L , Tian Y , et al. Novel susceptibility loci for moyamoya disease revealed by a genome‐wide association study. Stroke. 2018;49(1):11‐18. 29273593 10.1161/STROKEAHA.117.017430

[97] Park YS , Jeon YJ , Kim HS , et al. The roles of methylenetetrahydrofolate reductase 677C>T and 1298A>C polymorphisms in moyamoya disease patients. Child's Nerv Syst: ChNS. 2014;30(10):1687‐1695. 25098357 10.1007/s00381-014-2495-3

[98] Hervé D , Philippi A , Belbouab R , et al. Loss of α1β1 soluble guanylate cyclase, the major nitric oxide receptor, leads to moyamoya and achalasia. Am J Hum Genet. 2014;94(3):385‐394. 24581742 10.1016/j.ajhg.2014.01.018 PMC3951937

[99] Wallace S , Guo DC , Regalado E , et al. Disrupted nitric oxide signaling due to GUCY1A3 mutations increases risk for moyamoya disease, achalasia and hypertension. Clin Genet. 2016;90(4):351‐360. 26777256 10.1111/cge.12739 PMC4949143

[100] Zhang Q , Liu Y , Zhang D , et al. RNF213 as the major susceptibility gene for Chinese patients with moyamoya disease and its clinical relevance. J Neurosurg. 2017;126(4):1106‐1113. 27128593 10.3171/2016.2.JNS152173

[101] Miskinyte S , Butler MG , Hervé D , et al. Loss of BRCC3 deubiquitinating enzyme leads to abnormal angiogenesis and is associated with syndromic moyamoya. Am J Hum Genet. 2011;88(6):718‐728. 21596366 10.1016/j.ajhg.2011.04.017 PMC3113251

[102] Janczar S , Fogtman A , Koblowska M , et al. Novel severe hemophilia A and moyamoya (SHAM) syndrome caused by Xq28 deletions encompassing F8 and BRCC3 genes. Blood. 2014;123(25):4002‐4004. 24948625 10.1182/blood-2014-02-553685

[103] Lavin M , Jenkins PV , Keenan C , et al. X‐linked moyamoya syndrome associated with severe haemophilia A. Haemophilia. 2016;22(1):e51‐4. 26422091 10.1111/hae.12806

[104] Tzeravini E , Samara S , Kouramba A , et al. Severe hemophilia A and moyamoya syndrome in a 19‐year‐old boy caused by Xq28 microdeletion. Case Rep Neurol. 2022;14(2):261‐267. 35815106 10.1159/000524482 PMC9210018

[105] Kundishora AJ , Peters ST , Pinard A , et al. DIAPH1 variants in non‐East Asian patients with sporadic moyamoya disease. JAMA Neurol. 2021;78(8):993‐1003. 34125151 10.1001/jamaneurol.2021.1681 PMC8204259

[106] Kamada F , Aoki Y , Narisawa A , et al. A genome‐wide association study identifies RNF213 as the first moyamoya disease gene. J Hum Genet. 2011;56(1):34‐40. 21048783 10.1038/jhg.2010.132

[107] Morito D , Nishikawa K , Hoseki J , et al. Moyamoya disease‐associated protein mysterin/RNF213 is a novel AAA+ ATPase, which dynamically changes its oligomeric state. Sci Rep. 2014;4:4442. 24658080 10.1038/srep04442 PMC3963067

[108] Liu W , Hitomi T , Kobayashi H , Harada KH , Koizumi A . Distribution of moyamoya disease susceptibility polymorphism p.R4810K in RNF213 in East and Southeast Asian populations. Neurol Med Chir (Tokyo). 2012;52(5):299‐303. 22688066 10.2176/nmc.52.299

[109] Cao Y , Kobayashi H , Morimoto T , Kabata R , Harada KH , Koizumi A . Frequency of RNF213 p.R4810K, a susceptibility variant for moyamoya disease, and health characteristics of carriers in the Japanese population. Environ Health Prevent Med. 2016;21(5):387‐390. 10.1007/s12199-016-0549-8 PMC5305994 27365075

[110] Kim EH , Yum MS , Ra YS , et al. Importance of RNF213 polymorphism on clinical features and long‐term outcome in moyamoya disease. J Neurosurg. 2016;124(5):1221‐1227. 26430847 10.3171/2015.4.JNS142900

[111] Lee MJ , Chen YF , Fan PC , et al. Mutation genotypes of RNF213 gene from moyamoya patients in Taiwan. J Neurol Sci. 2015;353(1–2):161‐165. 25956231 10.1016/j.jns.2015.04.019

[112] Shoemaker LD , Clark MJ , Patwardhan A , et al. Disease variant landscape of a large multiethnic population of moyamoya patients by exome sequencing. G3 (Bethesda, MD). 2015;6(1):41‐49. 26530418 10.1534/g3.115.020321 PMC4704723

[113] Cecchi AC , Guo D , Ren Z , et al. RNF213 rare variants in an ethnically diverse population with moyamoya disease. Stroke. 2014;45(11):3200‐3207. 25278557 10.1161/STROKEAHA.114.006244 PMC4420622

[114] Okazaki S , Morimoto T , Kamatani Y , et al. Moyamoya disease susceptibility variant RNF213 p.R4810K increases the risk of ischemic stroke attributable to large‐artery atherosclerosis. Circulation. 2019;139(2):295‐298. 30615506 10.1161/CIRCULATIONAHA.118.038439

[115] Kobayashi H , Brozman M , Kyselová K , et al. RNF213 rare variants in Slovakian and Czech moyamoya disease patients. PLoS One. 2016;11(10):e0164759. 27736983 10.1371/journal.pone.0164759 PMC5063318

[116] Koizumi A , Kobayashi H , Hitomi T , Harada KH , Habu T , Youssefian S . A new horizon of moyamoya disease and associated health risks explored through RNF213. Environ Health Prevent Med. 2016;21(2):55‐70. 10.1007/s12199-015-0498-7 PMC4771639 26662949

[117] Wang Y , Zhang Z , Wei L , et al. Predictive role of heterozygous p.R4810K of RNF213 in the phenotype of Chinese moyamoya disease. Neurology. 2020;94(7):e678‐e686. 31949090 10.1212/WNL.0000000000008901 PMC7176299

[118] Miyatake S , Touho H , Miyake N , et al. Sibling cases of moyamoya disease having homozygous and heterozygous c.14576G>A variant in RNF213 showed varying clinical course and severity. J Hum Genet. 2012;57(12):804‐806. 22931863 10.1038/jhg.2012.105

[119] Wang X , Zhang Z , Wang Y , et al. Clinical and genetic risk factors of long‐term outcomes after encephaloduroarteriosynangiosis in moyamoya disease in China. J Stroke Cerebrovasc Dis. 2021;30(7):105847. 33992965 10.1016/j.jstrokecerebrovasdis.2021.105847

[120] Ge P , Ye X , Liu X , et al. Association between p.R4810K variant and long‐term clinical outcome in patients with moyamoya disease. Front Neurol. 2019;10:662. 31293503 10.3389/fneur.2019.00662 PMC6603092

[121] Miyatake S , Miyake N , Touho H , et al. Homozygous c.14576G>A variant of RNF213 predicts early‐onset and severe form of moyamoya disease. Neurology. 2012;78(11):803‐810. 22377813 10.1212/WNL.0b013e318249f71f

[122] Kim WH , Kim SD , Nam MH , et al. Posterior circulation involvement and collateral flow pattern in moyamoya disease with the RNF213 polymorphism. Child's Nerv Syst: ChNS. 2019;35(2):309‐314. 30283986 10.1007/s00381-018-3985-5

[123] Sonobe S , Fujimura M , Niizuma K , et al. Temporal profile of the vascular anatomy evaluated by 9.4‐T magnetic resonance angiography and histopathological analysis in mice lacking RNF213: a susceptibility gene for moyamoya disease. Brain Res. 2014;1552:64‐71. 24440776 10.1016/j.brainres.2014.01.011

[124] Ahel J , Lehner A , Vogel A , et al. Moyamoya disease factor RNF213 is a giant E3 ligase with a dynein‐like core and a distinct ubiquitin‐transfer mechanism. eLife. 2020;9:e56185. 32573437 10.7554/eLife.56185 PMC7311170

[125] He S , Ye X , Duan R , et al. Epigenome‐wide association study reveals differential methylation sites and association of gene expression regulation with ischemic moyamoya disease in adults. Oxid Med Cell Longev. 2022;2022:7192060. 35368875 10.1155/2022/7192060 PMC8970806

[126] Wu Z , Jiang H , Zhang L , et al. Molecular analysis of RNF213 gene for moyamoya disease in the Chinese Han population. PLoS One. 2012;7(10):e48179. 23110205 10.1371/journal.pone.0048179 PMC3479116

[127] Harel T , Posey JE , Graham BH , et al. Atypical presentation of moyamoya disease in an infant with a de novo RNF213 variant. Am J Med Genet Part A. 2015;167a(11):2742‐2747. 26198278 10.1002/ajmg.a.37230 PMC4639746

[128] Guey S , Kraemer M , Hervé D , et al. Rare RNF213 variants in the C‐terminal region encompassing the RING‐finger domain are associated with moyamoya angiopathy in Caucasians. Eur J Hum Genet: EJHG. 2017;25(8):995‐1003. 28635953 10.1038/ejhg.2017.92 PMC5567158

[129] Butler GS , Overall CM . Updated biological roles for matrix metalloproteinases and new “intracellular” substrates revealed by degradomics. Biochemistry. 2009;48(46):10830‐10845. 19817485 10.1021/bi901656f

[130] Georgescu MM , Pinho Mda C , Richardson TE , et al. The defining pathology of the new clinical and histopathologic entity ACTA2‐related cerebrovascular disease. Acta Neuropathol Commun. 2015;3:81. 26637293 10.1186/s40478-015-0262-7 PMC4670506

[131] Shimada A , Nyitrai M , Vetter IR , et al. The core FH2 domain of diaphanous‐related formins is an elongated actin binding protein that inhibits polymerization. Mol Cell. 2004;13(4):511‐522. 14992721 10.1016/s1097-2765(04)00059-0

[132] Goode BL , Eck MJ . Mechanism and function of formins in the control of actin assembly. Annu Rev Biochem. 2007;76:593‐627. 17373907 10.1146/annurev.biochem.75.103004.142647

[133] Pan J , Lordier L , Meyran D , et al. The formin DIAPH1 (mDia1) regulates megakaryocyte proplatelet formation by remodeling the actin and microtubule cytoskeletons. Blood. 2014;124(26):3967‐3977. 25298036 10.1182/blood-2013-12-544924

[134] Stritt S , Nurden P , Turro E , et al. A gain‐of‐function variant in DIAPH1 causes dominant macrothrombocytopenia and hearing loss. Blood. 2016;127(23):2903‐2914. 26912466 10.1182/blood-2015-10-675629

[135] Gaengel K , Genové G , Armulik A , Betsholtz C . Endothelial‐mural cell signaling in vascular development and angiogenesis. Arterioscler Thromb Vasc Biol. 2009;29(5):630‐638. 19164813 10.1161/ATVBAHA.107.161521

[136] Rafat N , Beck G , Peña‐Tapia PG , Schmiedek P , Vajkoczy P . Increased levels of circulating endothelial progenitor cells in patients with moyamoya disease. Stroke. 2009;40(2):432‐438. 19095988 10.1161/STROKEAHA.108.529420

[137] Müller CR , Ehninger G , Goldmann SF . Gene and haplotype frequencies for the loci hLA‐A, hLA‐B, and hLA‐DR based on over 13,000 German blood donors. Hum Immunol. 2003;64(1):137‐151. 12507825 10.1016/s0198-8859(02)00706-1

[138] Shiina T , Hosomichi K , Inoko H , Kulski JK . The HLA genomic loci map: expression, interaction, diversity and disease. J Hum Genet. 2009;54(1):15‐39. 19158813 10.1038/jhg.2008.5

[139] Moncada S , Higgs A . The L‐arginine‐nitric oxide pathway. New Engl J Med. 1993;329(27):2002‐2012. 7504210 10.1056/NEJM199312303292706

[140] Moro MA , Cárdenas A , Hurtado O , Leza JC , Lizasoain I . Role of nitric oxide after brain ischaemia. Cell Calcium. 2004;36(3–4):265‐275. 15261482 10.1016/j.ceca.2004.02.011

[141] Loscalzo J . Homocysteine‐mediated thrombosis and angiostasis in vascular pathobiology. J Clin Invest. 2009;119(11):3203‐3205. 19841539 10.1172/JCI40924 PMC2769170

[142] Ge P , Zhang Q , Ye X , et al. Modifiable risk factors associated with moyamoya disease: a case‐control study. Stroke. 2020;51(8):2472‐2479. 32640948 10.1161/STROKEAHA.120.030027

[143] Ueland PM , Hustad S , Schneede J , Refsum H , Vollset SE . Biological and clinical implications of the MTHFR C677T polymorphism. Trends Pharmacol Sci. 2001;22(4):195‐201. 11282420 10.1016/s0165-6147(00)01675-8

[144] Weisberg I , Tran P , Christensen B , Sibani S , Rozen R . A second genetic polymorphism in methylenetetrahydrofolate reductase (MTHFR) associated with decreased enzyme activity. Mol Genet Metab. 1998;64(3):169‐172. 9719624 10.1006/mgme.1998.2714

[145] He Q , Ge P , Ye X , et al. Hyperhomocysteinemia is a predictor for poor postoperative angiogenesis in adult patients with moyamoya disease. Front Neurol. 2022;13:902474. 35720075 10.3389/fneur.2022.902474 PMC9201052

[146] Mineharu Y , Miyamoto S . RNF213 and GUCY1A3 in moyamoya disease: key regulators of metabolism, inflammation, and vascular stability. Front Neurol. 2021;12:687088. 34381413 10.3389/fneur.2021.687088 PMC8350054

[147] Mattei AL , Bailly N , Meissner A . DNA methylation: a historical perspective. Trends Genet. 2022;38(7):676‐707. 35504755 10.1016/j.tig.2022.03.010

[148] Krishna Smriti M , Trollope Alexandra F , Golledge J . The relevance of epigenetics to occlusive cerebral and peripheral arterial disease. Clin Sci. 2015;128(9):537‐558. 10.1042/CS20140491 25671777

[149] Smith ZD , Hetzel S , Meissner A . DNA methylation in mammalian development and disease. Nat Rev Genet. 2024;26(1):7‐30. 39134824 10.1038/s41576-024-00760-8

[150] Wang Y , Zhang L , Lyu T , et al. Association of DNA methylation/demethylation with the functional outcome of stroke in a hyperinflammatory state. Neural Regen Res. 2024;19(10):2229‐2239. 38488557 10.4103/1673-5374.392890 PMC11034580

[151] Golledge J , Biros E , Bingley J , Iyer V , Krishna SM . Epigenetics and peripheral artery disease. Curr Atheroscler Rep. 2016;18(4):15. 26888065 10.1007/s11883-016-0567-4

[152] Endres M , Meisel A , Biniszkiewicz D , et al. DNA methyltransferase contributes to delayed ischemic brain injury. J Neurosci. 2000;20(9):3175‐3181. 10777781 10.1523/JNEUROSCI.20-09-03175.2000 PMC6773114

[153] Xu S , Chen T , Yu J , et al. Insights into the regulatory role of epigenetics in moyamoya disease: current advances and future prospectives. Mol Ther—Nucleic Acids. 2024;35(3):102281. 39188306 10.1016/j.omtn.2024.102281 PMC11345382

[154] Sung HY , Lee JY , Park AK , et al. Aberrant promoter hypomethylation of Sortilin 1: a moyamoya disease biomarker. J Stroke. 2018;20(3):350‐361. 30309230 10.5853/jos.2018.00962 PMC6186926

[155] He S , Ye X , Duan R , et al. Epigenome‐wide association study reveals differential methylation sites and association of gene expression regulation with ischemic moyamoya disease in adults. Oxid Med Cell Longev. 2022;2022:1‐13. 10.1155/2022/7192060 PMC8970806 35368875

[156] Tokairin K , Ito M , Lee AG , et al. Genome‐wide DNA methylation profiling reveals low methylation variability in moyamoya disease. Transl Stroke Res. 2024; 2024. 10.1007/s12975-024-01299-w PMC12202675 39356405

[157] Asselman C , Hemelsoet D , Eggermont D , Dermaut B , Impens F . Moyamoya disease emerging as an immune‐related angiopathy. Trends Mol Med. 2022;28(11):939‐950. 36115805 10.1016/j.molmed.2022.08.009

[158] Masuda J , Ogata J , Yutani C . Smooth muscle cell proliferation and localization of macrophages and T cells in the occlusive intracranial major arteries in moyamoya disease. Stroke. 1993;24(12):1960‐1967. 7902623 10.1161/01.str.24.12.1960

[159] Lin R , Xie Z , Zhang J , et al. Clinical and immunopathological features of moyamoya disease. PLoS One. 2012;7(4):e36386. 22558457 10.1371/journal.pone.0036386 PMC3338675

[160] Sigdel TK , Shoemaker LD , Chen R , et al. Immune response profiling identifies autoantibodies specific to moyamoya patients. Orphanet J Rare Dis. 2013;8:45. 23518061 10.1186/1750-1172-8-45 PMC3648437

[161] Fujimura M , Fujimura T , Kakizaki A , et al. Increased serum production of soluble CD163 and CXCL5 in patients with moyamoya disease: involvement of intrinsic immune reaction in its pathogenesis. Brain Res. 2018;1679:39‐44. 29174692 10.1016/j.brainres.2017.11.013

[162] Guo L , Akahori H , Harari E , et al. CD163+ macrophages promote angiogenesis and vascular permeability accompanied by inflammation in atherosclerosis. J Clin Investig. 2018;128(3):1106‐1124. 29457790 10.1172/JCI93025 PMC5824873

[163] Peng W , Xie Y , Liu Y , et al. Targeted delivery of CD163+ macrophage‐derived small extracellular vesicles via RGD peptides promote vascular regeneration and stabilization after spinal cord injury. J Control Release. 2023;361:750‐765. 37586563 10.1016/j.jconrel.2023.08.025

[164] Ni G , Liu W , Huang X , et al. Increased levels of circulating SDF‐1α and CD34+ CXCR4+ cells in patients with moyamoya disease. Eur J Neurol. 2011;18(11):1304‐1309. 21435112 10.1111/j.1468-1331.2011.03393.x

[165] Yoshihara T , Taguchi A , Matsuyama T , et al. Increase in circulating CD34‐positive cells in patients with angiographic evidence of moyamoya‐like vessels. J Cereb Blood Flow Metab. 2008;28(6):1086‐1089. 18231114 10.1038/jcbfm.2008.1

[166] Weng L , Cao X , Han L , et al. Association of increased Treg and Th17 with pathogenesis of moyamoya disease. Sci Rep. 2017;7(1):3071. 28596558 10.1038/s41598-017-03278-8 PMC5465197

[167] Kanoke A , Fujimura M , Niizuma K , et al. Temporal profile of magnetic resonance angiography and decreased ratio of regulatory T cells after immunological adjuvant administration to mice lacking RNF213, a susceptibility gene for moyamoya disease. Brain Res. 2016;1642:1‐9. 26972532 10.1016/j.brainres.2016.03.009

[168] Lužnik Z , Anchouche S , Dana R , Yin J . Regulatory T cells in angiogenesis. J Immunol. 2020;205(10):2557‐2565. 33168598 10.4049/jimmunol.2000574 PMC7664842

[169] Mejia‐Munne JC , Ellis JA , Feldstein NA , Meyers PM , Connolly ES . Moyamoya and inflammation. World Neurosurg. 2017;100:575‐578. 28093343 10.1016/j.wneu.2017.01.012

[170] Mikami T , Suzuki H , Komatsu K , Mikuni N . Influence of inflammatory disease on the pathophysiology of moyamoya disease and quasi‐moyamoya disease. Neurol Med Chir (Tokyo). 2019;59(10):361‐370. 31281171 10.2176/nmc.ra.2019-0059 PMC6796064

[171] Shirozu N , Ohgidani M , Hata N , et al. Angiogenic and inflammatory responses in human induced microglia‐like (iMG) cells from patients with moyamoya disease. Sci Rep. 2023;13(1):14842. 37684266 10.1038/s41598-023-41456-z PMC10491754

[172] Peruzzotti‐Jametti L , Willis CM , Krzak G , et al. Mitochondrial complex I activity in microglia sustains neuroinflammation. Nature. 2024;628(8006):195‐203. 38480879 10.1038/s41586-024-07167-9 PMC10990929

[173] Huang D , Qi H , Yang H , Chen M . Plasma exosomal microRNAs are non‐invasive biomarkers of moyamoya disease: a pilot study. Clinics (Sao Paulo). 2023;78:100247. 37413774 10.1016/j.clinsp.2023.100247 PMC10344806

[174] Sudhir BJ , Keelara AG , Venkat EH , Kazumata K , Sundararaman A . The mechanobiological theory: a unifying hypothesis on the pathogenesis of moyamoya disease based on a systematic review. Neurosurg Focus. 2021;51(3):E6. 10.3171/2021.6.FOCUS21281 34469862

[175] Kang K , Shen Y , Zhang Q , et al. MicroRNA expression in circulating leukocytes and bioinformatic analysis of patients with moyamoya disease. Front Genet. 2022;13:816919. 35669195 10.3389/fgene.2022.816919 PMC9163834

[176] Blaj LA , Cucu AI , Tamba BI , Turliuc MD . The role of the NF‐kB pathway in intracranial aneurysms. Brain Sci. 2023;13(12):1660. 38137108 10.3390/brainsci13121660 PMC10871091

[177] Yuan S , Liu H , Yuan D , et al. PNPLA3 I148M mediates the regulatory effect of NF‐kB on inflammation in PA‐treated HepG2 cells. J Cell Mol Med. 2020;24(2):1541‐1552. 31793207 10.1111/jcmm.14839 PMC6991629

[178] Baeriswyl DC , Prionisti I , Peach T , et al. Disturbed flow induces a sustained, stochastic NF‐kappaB activation which may support intracranial aneurysm growth in vivo. Sci Rep. 2019;9(1):4738. 30894565 10.1038/s41598-019-40959-y PMC6426999

[179] Takeda M , Tezuka T , Kim M , et al. Moyamoya disease patient mutations in the RING domain of RNF213 reduce its ubiquitin ligase activity and enhance NFkappaB activation and apoptosis in an AAA+ domain‐dependent manner. Biochem Biophys Res Commun. 2020;525(3):668‐674. 32139119 10.1016/j.bbrc.2020.02.024

[180] Xu Y , Chen B , Guo Z , et al. Identification of diagnostic markers for moyamoya disease by combining bulk RNA‐sequencing analysis and machine learning. Sci Rep. 2024;14(1):5931. 38467737 10.1038/s41598-024-56367-w PMC10928210

[181] Xin P , Xu X , Deng C , et al. The role of JAK/STAT signaling pathway and its inhibitors in diseases. Int Immunopharmacol. 2020;80:106210. 31972425 10.1016/j.intimp.2020.106210

[182] Murphy ES , Xie H , Merchant TE , Yu JS , Chao ST , Suh JH . Review of cranial radiotherapy‐induced vasculopathy. J Neurooncol. 2015;122(3):421‐429. 25670390 10.1007/s11060-015-1732-2

[183] Desai SS , Paulino AC , Mai WY , Teh BS . Radiation‐induced moyamoya syndrome. Int J Radiat Oncol Biol Phys. 2006;65(4):1222‐1227. 16626890 10.1016/j.ijrobp.2006.01.038

[184] Coderre JA , Morris GM , Micca PL , et al. Late effects of radiation on the central nervous system: role of vascular endothelial damage and glial stem cell survival. Radiat Res. 2006;166(3):495‐503. 16953668 10.1667/RR3597.1

[185] Reinhold HSCW , Hopewell JW , van der Berg AP . Development of blood vessel‐related radiation damage in the fimbria of the central nervous system. Int J Radiat Oncol Biol Phys. 1990;18(1):37‐42. 2298633 10.1016/0360-3016(90)90264-k

[186] Manion B , Sung WS . Radiation‐induced moyamoya disease after childhood astrocytoma. J Clin Neurosci. 2011;18(10):1403‐1405. 21783367 10.1016/j.jocn.2011.01.032

[187] Scala M , Fiaschi P , Cama A , et al. Radiation‐induced moyamoya syndrome in children with brain tumors: case series and literature review. World Neurosurg. 2020;135:118‐129. 31805403 10.1016/j.wneu.2019.11.155

[188] Mehmood Qadri H , Bashir RA , Amir A , et al. Post‐infectious moyamoya syndrome: a review of existing scientific literature from 2000 to 2023. Cureus. 2024;16(7):e63643. 39092349 10.7759/cureus.63643 PMC11292458

[189] Arficho KT , Gumma C , Chakko MN . Post cryptococcal moyamoya syndrome in adult human immunodeficiency virus patient with anterior and posterior circulation involvement: case report. Cureus. 2023;15(8):e44052. 37746378 10.7759/cureus.44052 PMC10517719

[190] Yamada H , Deguchi K , Tanigawara T , et al. The relationship between moyamoya disease and bacterial infection. Clin Neurol Neurosurg. 1997;99(suppl 2):S221‐S224. 9409442 10.1016/s0303-8467(97)00048-6

[191] Mineharu Y , Takagi Y , Koizumi A , et al. Genetic and nongenetic factors for contralateral progression of unilateral moyamoya disease: the first report from the SUPRA Japan Study Group. J Neurosurg. 2022;136(4):1005‐1014. 34507293 10.3171/2021.3.JNS203913

[192] Ohya Y , Matsuo R , Sato N , et al. Causes of ischemic stroke in young adults versus non‐young adults: a multicenter hospital‐based observational study. PLoS One. 2022;17(7):e0268481. 35830430 10.1371/journal.pone.0268481 PMC9278748

[193] McCarty JL , Leung LY , Peterson RB , et al. Ischemic infarction in young adults: a review for radiologists. Radiographics. 2019;39(6):1629‐1648. 31589580 10.1148/rg.2019190033

[194] Yu X , Ge P , Zhai Y , et al. Gut microbiota in adults with moyamoya disease: characteristics and biomarker identification. Front Cell Infect Microbiol. 2023;13:1252681. 37915847 10.3389/fcimb.2023.1252681 PMC10616959

[195] Vandereyken K , Sifrim A , Thienpont B , Voet T . Methods and applications for single‐cell and spatial multi‐omics. Nat Rev Genet. 2023;24(8):494‐515. 36864178 10.1038/s41576-023-00580-2 PMC9979144

[196] He S , Zhang J , Liu Z , et al. Upregulated cytoskeletal proteins promote pathological angiogenesis in moyamoya disease. Stroke. 2023;54(12):3153‐3164. 37886851 10.1161/STROKEAHA.123.044476

[197] He S , Wang Y , Liu Z , et al. Metabolomic signatures associated with pathological angiogenesis in moyamoya disease. Clin Transl Med. 2023;13(12):e1492. 38037492 10.1002/ctm2.1492 PMC10689969

[198] Nakamura Y , Mineharu Y , Kamata T , et al. Lack of association between seropositivity of vasculopathy‐related viruses and moyamoya disease. J Stroke Cerebrovasc Dis. 2022;31(7):106509. 35500358 10.1016/j.jstrokecerebrovasdis.2022.106509

[199] Li H , Cao X , Gu X , et al. GM‐CSF promotes the development of dysfunctional vascular networks in moyamoya disease. Neurosci Bull. 2023;40(4):451‐465. 38113014 10.1007/s12264-023-01158-y PMC11003948

[200] Kaku Y , Morioka M , Ohmori Y , et al. Outer‐diameter narrowing of the internal carotid and middle cerebral arteries in moyamoya disease detected on 3D constructive interference in steady‐state MR image: is arterial constrictive remodeling a major pathogenesis?. Acta Neurochir (Wien). 2012;154(12):2151‐2157. 22935819 10.1007/s00701-012-1472-4

[201] Sonobe S , Fujimura M , Niizuma K , et al. Temporal profile of the vascular anatomy evaluated by 9.4‐T magnetic resonance angiography and histopathological analysis in mice lacking RNF213: a susceptibility gene for moyamoya disease. Brain Res. 2014;1552:64‐71. 24440776 10.1016/j.brainres.2014.01.011

[202] Meisel A , Liu W , Morito D , et al. Identification of RNF213 as a susceptibility gene for moyamoya disease and its possible role in vascular development. PLoS One. 2011;6(7):e22542. 21799892 10.1371/journal.pone.0022542 PMC3140517

[203] Kamata I , Terai Y , Ohmoto T . Attempt to establish an experimental animal model of moyamoya disease using immuno‐embolic material–histological changes of the arterial wall resulting from immunological reaction in cats. Acta Med Okayama. 2003;57(3):143‐150. 12908012 10.18926/AMO/32831

[204] Tokairin K , Hamauchi S , Ito M , et al. Vascular smooth muscle cell derived from IPS cell of moyamoya disease—comparative characterization with endothelial cell transcriptome. J Stroke Cerebrovasc Dis. 2020;29(12):105305. 32992193 10.1016/j.jstrokecerebrovasdis.2020.105305

[205] Blecharz KG , Frey D , Schenkel T , et al. Autocrine release of angiopoietin‐2 mediates cerebrovascular disintegration in moyamoya disease. J Cereb Blood Flow Metab. 2016;37(4):1527‐1539. 27381827 10.1177/0271678X16658301 PMC5453470

[206] Wimmer RA , Leopoldi A , Aichinger M , et al. Human blood vessel organoids as a model of diabetic vasculopathy. Nature. 2019;565(7740):505‐510. 30651639 10.1038/s41586-018-0858-8 PMC7116578

[207] He S , Zhang J , Wang X , et al. Organoid modeling and single‐cell profiling uncover the migration mechanism of smooth muscle cells in moyamoya disease. bioRxiv. 2024.

[208] Fukui M . Guidelines for the diagnosis and treatment of spontaneous occlusion of the circle of Willis (‘moyamoya’ disease). Research Committee on Spontaneous Occlusion of the Circle of Willis (Moyamoya Disease) of the Ministry of Health and Welfare, Japan. Clin Neurol Neurosurg. 1997;99(suppl 2):S238‐S240. 9409446

[209] Research Committee on the Pathology and Treatment of Spontaneous Occlusion of the Circle of Willis; Health Labour Sciences Research Grant for Research on Measures for Infractable Diseases . Guidelines for diagnosis and treatment of moyamoya disease (spontaneous occlusion of the circle of Willis). Neurol Med Chir. 2012;52(5):245‐266. 10.2176/nmc.52.245 22870528

[210] Suzuki J , Takaku A . Cerebrovascular moyamoya disease. Disease showing abnormal net‐like vessels in base of brain. Arch Neurol. 1969;20(3):288‐299. 5775283 10.1001/archneur.1969.00480090076012

[211] Yamada I , Matsushima Y , Suzuki S . Moyamoya disease: diagnosis with three‐dimensional time‐of‐flight MR angiography. Radiology. 1992;184(3):773‐778. 1509066 10.1148/radiology.184.3.1509066

[212] Houkin K , Tanaka N , Takahashi A , Kamiyama H , Abe H , Kajii N . Familial occurrence of moyamoya disease. Magnetic resonance angiography as a screening test for high‐risk subjects. Childs Nerv Syst. 1994;10(7):421‐425. 7842430 10.1007/BF00303605

[213] Houkin K , Aoki T , Takahashi A , Abe H . Diagnosis of moyamoya disease with magnetic resonance angiography. Stroke. 1994;25(11):2159‐2164. 7974539 10.1161/01.str.25.11.2159

[214] Houkin K , Nakayama N , Kuroda S , Nonaka T , Shonai T , Yoshimoto T . Novel magnetic resonance angiography stage grading for moyamoya disease. Cerebrovasc Dis. 2005;20(5):347‐354. 16131804 10.1159/000087935

[215] Fujiwara H , Momoshima S , Kuribayashi S . Leptomeningeal high signal intensity (ivy sign) on fluid‐attenuated inversion‐recovery (FLAIR) MR images in moyamoya disease. Eur J Radiol. 2005;55(2):224‐230. 16036151 10.1016/j.ejrad.2004.11.009

[216] Ryoo S , Cha J , Kim SJ , et al. High‐resolution magnetic resonance wall imaging findings of moyamoya disease. Stroke. 2014;45(8):2457‐2460. 24947295 10.1161/STROKEAHA.114.004761

[217] Oh BH , Moon HC , Baek HM , et al. Comparison of 7T and 3T MRI in patients with moyamoya disease. Magn Reson Imaging. 2017;37:134‐138. 27899331 10.1016/j.mri.2016.11.019

[218] Togao O , Hiwatashi A , Obara M , et al. 4D ASL‐based MR angiography for visualization of distal arteries and leptomeningeal collateral vessels in moyamoya disease: a comparison of techniques. Eur Radiol. 2018;28(11):4871‐4881. 29737389 10.1007/s00330-018-5462-7

[219] Funaki T , Takahashi JC , Yoshida K , et al. Periventricular anastomosis in moyamoya disease: detecting fragile collateral vessels with MR angiography. J Neurosurg. 2016;124(6):1766‐1772. 26613176 10.3171/2015.6.JNS15845

[220] Funaki T , Takahashi JC , Houkin K , et al. High rebleeding risk associated with choroidal collateral vessels in hemorrhagic moyamoya disease: analysis of a nonsurgical cohort in the Japan Adult Moyamoya Trial. J Neurosurg. 2019;130(2):525‐530. 29498573 10.3171/2017.9.JNS17576

[221] Schmiedek P , Piepgras A , Leinsinger G , Kirsch CM , Einhüpl K . Improvement of cerebrovascular reserve capacity by EC‐IC arterial bypass surgery in patients with ICA occlusion and hemodynamic cerebral ischemia. J Neurosurg. 1994;81(2):236‐244. 8027807 10.3171/jns.1994.81.2.0236

[222] Yonas H , Smith HA , Durham SR , Pentheny SL , Johnson DW . Increased stroke risk predicted by compromised cerebral blood flow reactivity. J Neurosurg. 1993;79(4):483‐489. 8410214 10.3171/jns.1993.79.4.0483

[223] Gupta A , Chazen JL , Hartman M , et al. Cerebrovascular reserve and stroke risk in patients with carotid stenosis or occlusion: a systematic review and meta‐analysis. Stroke. 2012;43(11):2884‐2891. 23091119 10.1161/STROKEAHA.112.663716 PMC3500140

[224] Czabanka M , Peña‐Tapia P , Schubert GA , et al. Proposal for a new grading of moyamoya disease in adult patients. Cerebrovasc Dis (Basel, Switzerland). 2011;32(1):41‐50. 10.1159/000326077 21576942

[225] Ikezaki K , Matsushima T , Kuwabara Y , Suzuki SO , Nomura T , Fukui M . Cerebral circulation and oxygen metabolism in childhood moyamoya disease: a perioperative positron emission tomography study. J Neurosurg. 1994;81(6):843‐850. 7965114 10.3171/jns.1994.81.6.0843

[226] Nariai T , Matsushima Y , Imae S , et al. Severe haemodynamic stress in selected subtypes of patients with moyamoya disease: a positron emission tomography study. J Neurol Neurosurg Psychiatry. 2005;76(5):663‐669. 15834024 10.1136/jnnp.2003.025049 PMC1739646

[227] Hara S , Kudo T , Hayashi S , et al. Improvement in cognitive decline after indirect bypass surgery in adult moyamoya disease: implication of (15)O‐gas positron emission tomography. Ann Nucl Med. 2020;34(7):467‐475. 32378149 10.1007/s12149-020-01473-8

[228] Roder C , Haas P , Fudali M , et al. Neuropsychological impairment in adults with moyamoya angiopathy: preoperative assessment and correlation to MRI and H(2)(15)O PET. Neurosurg Rev. 2020;43(6):1615‐1622. 31728848 10.1007/s10143-019-01192-3

[229] Touho H , Karasawa J , Ohnishi H . Preoperative and postoperative evaluation of cerebral perfusion and vasodilatory capacity with 99mTc‐HMPAO SPECT and acetazolamide in childhood moyamoya disease. Stroke. 1996;27(2):282‐289. 8571424 10.1161/01.str.27.2.282

[230] Saito N , Nakagawara J , Nakamura H , Teramoto A . Assessment of cerebral hemodynamics in childhood moyamoya disease using a quantitative and a semiquantitative IMP‐SPECT study. Ann Nucl Med. 2004;18(4):323‐331. 15359926 10.1007/BF02984471

[231] So Y , Lee H‐Y , Kim S‐K , et al. Prediction of the clinical outcome of pediatric moyamoya disease with postoperative basal/acetazolamide stress brain perfusion SPECT after revascularization surgery. Stroke. 2005;36(7):1485‐1489. 15947261 10.1161/01.STR.0000170709.95185.b1

[232] Horowitz M , Yonas H , Albright AL . Evaluation of cerebral blood flow and hemodynamic reserve in symptomatic moyamoya disease using stable xenon‐CT blood flow. Surg Neurol. 1995;44(3):251‐261. discussion 262. 8545777 10.1016/0090-3019(95)00188-3

[233] Nambu K , Suzuki R , Hirakawa K . Cerebral blood flow: measurement with xenon‐enhanced dynamic helical CT. Radiology. 1995;195(1):53‐57. 7892495 10.1148/radiology.195.1.7892495

[234] Suzuki R , Nariai T , Matsushima Y , Hirakawa K . Xe‐CT in cerebrovascular disease and moyamoya disease. Acta Neurol Scand Suppl. 1996;166:69‐71. 8686446 10.1111/j.1600-0404.1996.tb00552.x

[235] McAuley DJ , Poskitt K , Steinbok P . Predicting stroke risk in pediatric moyamoya disease with xenon‐enhanced computed tomography. Neurosurgery. 2004;55(2):327‐332. discussion 332–333. 15271238 10.1227/01.neu.0000129695.91536.41

[236] Sakamoto S , Ohba S , Shibukawa M , Kiura Y , Arita K , Kurisu K . CT perfusion imaging for childhood moyamoya disease before and after surgical revascularization. Acta Neurochir (Wien). 2006;148(1):77‐81. discussion 81. 16184319 10.1007/s00701-005-0634-z

[237] Kang KH , Kim HS , Kim SY . Quantitative cerebrovascular reserve measured by acetazolamide‐challenged dynamic CT perfusion in ischemic adult moyamoya disease: initial experience with angiographic correlation. AJNR Am J Neuroradiol. 2008;29(8):1487‐1493. 18499785 10.3174/ajnr.A1129 PMC8119064

[238] Rim NJ , Kim HS , Shin YS , Kim SY . Which CT perfusion parameter best reflects cerebrovascular reserve?: correlation of acetazolamide‐challenged CT perfusion with single‐photon emission CT in moyamoya patients. AJNR Am J Neuroradiol. 2008;29(9):1658‐1663. 18617583 10.3174/ajnr.A1229 PMC8118762

[239] Ishii Y , Nariai T , Tanaka Y , et al. Practical clinical use of dynamic susceptibility contrast magnetic resonance imaging for the surgical treatment of moyamoya disease. Neurosurgery. 2014;74(3):302‐309. 24335813 10.1227/NEU.0000000000000266

[240] Ishii Y , Tanaka Y , Momose T , et al. Chronologic evaluation of cerebral hemodynamics by dynamic susceptibility contrast magnetic resonance imaging after indirect bypass surgery for moyamoya disease. World Neurosurg. 2017;108:427‐435. 28893695 10.1016/j.wneu.2017.09.001

[241] Wolf RL , Detre JA . Clinical neuroimaging using arterial spin‐labeled perfusion magnetic resonance imaging. Neurotherapeutics. 2007;4(3):346‐359. 17599701 10.1016/j.nurt.2007.04.005 PMC2031222

[242] Yun TJ , Sohn C‐H , Han MH , et al. Effect of delayed transit time on arterial spin labeling: correlation with dynamic susceptibility contrast perfusion magnetic resonance in moyamoya disease. Investig Radiol. 2013;48(11):795‐802. 23764569 10.1097/RLI.0b013e3182981137

[243] Blauwblomme T , Lemaitre H , Naggara O , et al. Cerebral blood flow improvement after indirect revascularization for pediatric moyamoya disease: a statistical analysis of arterial spin‐labeling MRI. AJNR Am J Neuroradiol. 2016;37(4):706‐712. 26585258 10.3174/ajnr.A4592 PMC7960163

[244] Ha JY , Choi YH , Lee S , et al. Arterial spin labeling MRI for quantitative assessment of cerebral perfusion before and after cerebral revascularization in children with moyamoya disease. Korean J Radiol. 2019;20(6):985‐996. 31132824 10.3348/kjr.2018.0651 PMC6536794

[245] Quon JL , Kim LH , Lober RM , Maleki M , Steinberg GK , Yeom KW . Arterial spin‐labeling cerebral perfusion changes after revascularization surgery in pediatric moyamoya disease and syndrome. J Neurosurg Pediatr. 2019;23(4):486‐492. 30738390 10.3171/2018.11.PEDS18498

[246] Agarwal V , Singh P , Ahuja CK , Gupta SK , Aggarwal A , Narayanan R . Non‐invasive assessment of cerebral microvascular changes for predicting postoperative cerebral hyperperfusion after surgical revascularisation for moyamoya disease: an arterial spin labelling MRI study. Neuroradiology. 2021;63(4):563‐572. 33098435 10.1007/s00234-020-02583-w

[247] Muttaqin Z , Ohba S , Arita K , et al. Cerebral circulation in moyamoya disease: a clinical study using transcranial Doppler sonography. Surg Neurol. 1993;40(4):306‐313. 8211641 10.1016/0090-3019(93)90142-n

[248] Laborde G , Harders A , Klimek L , Hardenack M . Correlation between clinical, angiographic and transcranial Doppler sonographic findings in patients with moyamoya disease. Neurol Res. 1993;15(2):87‐92. 8099214 10.1080/01616412.1993.11740115

[249] Lee Y‐S , Jung K‐H , Roh J‐K . Diagnosis of moyamoya disease with transcranial Doppler sonography: correlation study with magnetic resonance angiography. J Neuroimag. 2004;14(4):319‐323. 10.1177/1051228404264958 15358951

[250] Ju K , Zhong L , Ni X , Cao H , Cheng G , Ding L . Cerebral vasomotor reactivity predicts the development of acute stroke in patients with internal carotid artery stenosis. Neurol Neurochir Pol. 2018;52(3):374‐378. 29361283 10.1016/j.pjnns.2017.12.015

[251] Yeh S‐J , Tang S‐C , Tsai L‐K , et al. Ultrasonographic changes after indirect revascularization surgery in pediatric patients with moyamoya disease. Ultrasound Med Biol. 2016;42(12):2844‐2851. 27639432 10.1016/j.ultrasmedbio.2016.07.016

[252] Yeh S‐J , Tang S‐C , Tsai L‐K , et al. Greater ultrasonographic changes in pediatric moyamoya patients compared with adults after indirect revascularization surgeries. J Neurosurg Pediatr. 2018;22(6):663‐671. 30168733 10.3171/2018.6.PEDS18151

[253] Adla T , Adlova R . Multimodality imaging of carotid stenosis. Int J Angiol. 2015;24(3):179‐184. 26417185 10.1055/s-0035-1556056 PMC4572013

[254] Du L , Jiang H , Li J , Duan T , Zhou C , Yan F . Imaging methods for surgical revascularization in patients with moyamoya disease: an updated review. Neurosurg Rev. 2022;45(1):343‐356. 34417671 10.1007/s10143-021-01596-0 PMC8827314

[255] Sam K , Poublanc J , Sobczyk O , et al. Assessing the effect of unilateral cerebral revascularisation on the vascular reactivity of the non‐intervened hemisphere: a retrospective observational study. BMJ Open. 2015;5(2):e006014. 10.1136/bmjopen-2014-006014 PMC4325130 25673438

[256] Rosen C , McKetton L , Russell J , et al. Long‐term changes in cerebrovascular reactivity following EC‐IC bypass for intracranial steno‐occlusive disease. J Clin Neurosci. 2018;54:77‐82. 29907385 10.1016/j.jocn.2018.06.009

[257] Dlamini N , Shah‐Basak P , Leung J , et al. Breath‐hold blood oxygen level‐dependent MRI: a tool for the assessment of cerebrovascular reserve in children with moyamoya disease. AJNR A J Neuroradiol. 2018;39(9):1717‐1723. 10.3174/ajnr.A5739 PMC7655282 30139753

[258] Hauser T‐K , Seeger A , Bender B , et al. Hypercapnic BOLD MRI compared to H(2)(15)O PET/CT for the hemodynamic evaluation of patients with moyamoya disease. Neuroimage Clin. 2019;22:101713. 30743136 10.1016/j.nicl.2019.101713 PMC6370561

[259] Liu P , Li Y , Pinho M , Park DC , Welch BG , Lu H . Cerebrovascular reactivity mapping without gas challenges. NeuroImage. 2017;146:320‐326. 27888058 10.1016/j.neuroimage.2016.11.054 PMC5321860

[260] Liu P , Liu G , Pinho MC , et al. Cerebrovascular reactivity mapping using resting‐state BOLD functional MRI in healthy adults and patients with moyamoya disease. Radiology. 2021;299(2):419‐425. 33687287 10.1148/radiol.2021203568 PMC8108558

[261] Lee M , Guzman R , Bell‐Stephens T , Steinberg GK . Intraoperative blood flow analysis of direct revascularization procedures in patients with moyamoya disease. J Cereb Blood Flow Metab. 2011;31(1):262‐274. 20588321 10.1038/jcbfm.2010.85 PMC3049490

[262] Yu Z , Xe Shi , Brohi SR , Qian H , Liu F , Yang Y . Measurement of blood flow in an intracranial artery bypass from the internal maxillary artery by intraoperative duplex sonography. J Ultrasound Med. 2017;36(2):439‐447. 28026888 10.7863/ultra.16.02011

[263] Yu Z , Yang Y , Xe Shi , Qian H , Liu F . A comparison of haemodynamics between subcranial‐intracranial bypass and the traditional extracranial‐intracranial bypass. Br J Neurosurg. 2017;31(6):668‐671. 28490201 10.1080/02688697.2017.1327015

[264] Morisawa H , Kawamata T , Kawashima A , et al. Hemodynamics and changes after STA‐MCA anastomosis in moyamoya disease and atherosclerotic cerebrovascular disease measured by micro‐Doppler ultrasonography. Neurosurg Rev. 2013;36(3):411‐419. 23192651 10.1007/s10143-012-0441-y

[265] Nomura M , Tamase A , Kamide T , et al. Pin‐point selection of recipient MCA at M4 for STA‐MCA bypass using micro‐Doppler ultrasonography. J Neurosurg Sci. 2017;61(4):446‐449. 28555489 10.23736/S0390-5616.16.03381-6

[266] Ye X , Liu X‐J , Ma L , et al. Clinical values of intraoperative indocyanine green fluorescence video angiography with Flow 800 software in cerebrovascular surgery. Chin Med J. 2013;126(22):4232‐4237. 24238503

[267] Yang T , Higashino Y , Kataoka H , et al. Correlation between reduction in microvascular transit time after superficial temporal artery‐middle cerebral artery bypass surgery for moyamoya disease and the development of postoperative hyperperfusion syndrome. J Neurosurg. 2018;128(5):1304‐1310. 28498060 10.3171/2016.11.JNS162403

[268] Uda K , Araki Y , Muraoka S , et al. Intraoperative evaluation of local cerebral hemodynamic change by indocyanine green videoangiography: prediction of incidence and duration of postoperative transient neurological events in patients with moyamoya disease. J Neurosurg. 2018(4):1‐9. 10.3171/2017.10.JNS171523 29676693

[269] Horie N , Fukuda Y , Izumo T , Hayashi K , Suyama K , Nagata I . Indocyanine green videoangiography for assessment of postoperative hyperperfusion in moyamoya disease. Acta Neurochir. 2014;156(5):919‐926. 24627037 10.1007/s00701-014-2054-4

[270] Machida T , Ono J , Nomura R , Fujikawa A , Nagano O , Higuchi Y . Venous reddening as a possible sign of hyperperfusion after superficial temporal artery‐middle cerebral artery anastomosis for moyamoya disease: case report. Neurol Med Chir (Tokyo). 2014;54(10):827‐831. 24670309 10.2176/nmc.cr.2013-0261 PMC4533381

[271] Machida T , Higuchi Y , Nakano S , et al. Cortical venous redness represents tissue circulation status in patients with moyamoya disease. Stroke. 2017;48(6):1665‐1667. 28446622 10.1161/STROKEAHA.116.015991

[272] Kearns KN , Sokolowski JD , Chadwell K , et al. The role of contrast‐enhanced ultrasound in neurosurgical disease. Neurosurg Focus. 2019;47(6):E8. 10.3171/2019.9.FOCUS19624 31786558

[273] Knieling F , Rüffer A , Cesnjevar R , et al. Transfontanellar contrast‐enhanced ultrasound for monitoring brain perfusion during neonatal heart surgery. Circ Cardiovasc Imaging. 2020;13(3):e010073. 32114827 10.1161/CIRCIMAGING.119.010073

[274] Premilovac D , Blackwood SJ , Ramsay CJ , Keske MA , Howells DW , Sutherland BA . Transcranial contrast‐enhanced ultrasound in the rat brain reveals substantial hyperperfusion acutely post‐stroke. J Cereb Blood Flow Metab. 2020;40(5):939‐953. 32063081 10.1177/0271678X20905493 PMC7181087

[275] Pang CH , Cho WS , Kang HS , Kim JE . Benefits and risks of antiplatelet medication in hemodynamically stable adult moyamoya disease. Sci Rep. 2021;11(1):19367. 34588601 10.1038/s41598-021-99009-1 PMC8481560

[276] Yamada S , Oki K , Itoh Y , et al. Effects of surgery and antiplatelet therapy in ten‐year follow‐up from the registry study of research committee on moyamoya disease in Japan. J Stroke Cerebrovasc Dis. 2016;25(2):340‐349. 26654669 10.1016/j.jstrokecerebrovasdis.2015.10.003

[277] Zhao Y , Zhang Q , Zhang D , Zhao Y . Effect of aspirin in postoperative management of adult ischemic moyamoya disease. World Neurosurg. 2017;105:728‐731. 28625901 10.1016/j.wneu.2017.06.057

[278] Seo WK , Kim JY , Choi EH , et al. Association of antiplatelet therapy, including cilostazol, with improved survival in patients with moyamoya disease in a nationwide study. J Am Heart Assoc. 2021;10(5):e017701. 33615836 10.1161/JAHA.120.017701 PMC8174237

[279] Onozuka D , Hagihara A , Nishimura K , et al. Prehospital antiplatelet use and functional status on admission of patients with non‐haemorrhagic moyamoya disease: a nationwide retrospective cohort study (J‐ASPECT study). BMJ Open. 2016;6(3):e009942. 10.1136/bmjopen-2015-009942 PMC4800148 27008684

[280] Chiba T , Setta K , Shimada Y , et al. Comparison of effects between clopidogrel and cilostazol on cerebral perfusion in nonsurgical adult patients with symptomatically ischemic moyamoya disease: subanalysis of a prospective cohort. J Stroke Cerebrovasc Dis. 2018;27(11):3373‐3379. 30174225 10.1016/j.jstrokecerebrovasdis.2018.07.041

[281] Aihara Y , Kashiwase S , Chiba K , et al. Aspirin use and platelet aggregation in ischemic onset‐type pediatric moyamoya patients with intractable headaches (moya‐ache). Child's Nerv Syst: ChNS. 2021;37(5):1649‐1657. 33404716 10.1007/s00381-020-04991-y

[282] Yeon JY , Kim JS , Hong SC . Incidental major artery aneurysms in patients with non‐hemorrhagic moyamoya disease. Acta Neurochir. 2011;153(6):1263‐1270. 21279660 10.1007/s00701-011-0948-y

[283] Kawaguchi S , Sakaki T , Morimoto T , Kakizaki T , Kamada K . Characteristics of intracranial aneurysms associated with moyamoya disease. A review of 111 cases. Acta Neurochir. 1996;138(11):1287‐1294. 8980731 10.1007/BF01411057

[284] Yu JL , Wang HL , Xu K , Li Y , Luo Q . Endovascular treatment of intracranial aneurysms associated with moyamoya disease or moyamoya syndrome. Interv Neuroradiol. 2010;16(3):240‐248. 20977854 10.1177/159101991001600302 PMC3277993

[285] Cho WS , Kim JE , Kim CH , et al. Long‐term outcomes after combined revascularization surgery in adult moyamoya disease. Stroke. 2014;45(10):3025‐3031. 25184359 10.1161/STROKEAHA.114.005624

[286] Miyamoto S , Yoshimoto T , Hashimoto N , et al. Effects of extracranial–intracranial bypass for patients with hemorrhagic moyamoya disease. Stroke. 2014;45(5):1415‐1421. 24668203 10.1161/STROKEAHA.113.004386

[287] Karasawa J , Kikuchi H , Furuse S , Kawamura J , Sakaki T . Treatment of moyamoya disease with STA‐MCA anastomosis. J Neurosurg. 1978;49(5):679‐688. 712390 10.3171/jns.1978.49.5.0679

[288] Nielsen TH , Abhinav K , Sussman ES , et al. Direct versus indirect bypass procedure for the treatment of ischemic moyamoya disease: results of an individualized selection strategy. J Neurosurg. 2020;134(5):1578‐1589. 32534489 10.3171/2020.3.JNS192847

[289] Houkin K , Kuroda S , Ishikawa T , Abe H . Neovascularization (angiogenesis) after revascularization in moyamoya disease. Which technique is most useful for moyamoya disease?. Acta Neurochir. 2000;142(3):269‐276. 10819257 10.1007/s007010050035

[290] Czabanka M , Pena‐Tapia P , Scharf J , et al. Characterization of direct and indirect cerebral revascularization for the treatment of European patients with moyamoya disease. Cerebrovasc Dis (Basel, Switzerland). 2011;32(4):361‐369. 10.1159/000330351 21921600

[291] Deng X , Gao F , Zhang D , et al. Direct versus indirect bypasses for adult ischemic‐type moyamoya disease: a propensity score‐matched analysis. J Neurosurg. 2018;128(6):1785‐1791. 28799875 10.3171/2017.2.JNS162405

[292] Ding JY , Shang SL , Sun ZS , et al. Remote ischemic conditioning for the treatment of ischemic moyamoya disease. CNS Neurosci Ther. 2020;26(5):549‐557. 31814317 10.1111/cns.13279 PMC7163773

[293] Kuroda S , Group AS . Asymptomatic moyamoya disease: literature review and ongoing AMORE study. Neurol Med Chir (Tokyo). 2015;55(3):194‐198. 25739434 10.2176/nmc.ra.2014-0305 PMC4533338

[294] Gross BA , Thomas AJ , Frerichs KU . Endovascular treatment of symptomatic moyamoya. Neurosurg Rev. 2014;37(4):579‐583. 24696002 10.1007/s10143-014-0542-x

[295] Nagamine Y , Takahashi S , Sonobe M . Multiple intracranial aneurysms associated with moyamoya disease. Case report. J Neurosurg. 1981;54(5):673‐676. 7229709 10.3171/jns.1981.54.5.0673

[296] Iwama T , Todaka T , Hashimoto N . Direct surgery for major artery aneurysm associated with moyamoya disease. Clin Neurol Neurosurg. 1997;99(suppl 2):S191‐S193. 9409435 10.1016/s0303-8467(97)00081-4

[297] Peltier J , Vinchon M , Soto‐Ares G , Dhellemmes P . Disappearance of a middle cerebral artery aneurysm associated with moyamoya syndrome after revascularization in a child: case report. Child's Nerv Syst: ChNS. 2008;24(12):1483‐1487. 18622621 10.1007/s00381-008-0670-0

[298] Satoh T , Yamamoto Y , Asari S , Sakurai M , Suzuki K . Disappearance and development of cerebral aneurysms in moyamoya disease. Case report. J Neurosurg. 1983;58(6):949‐953. 6854391 10.3171/jns.1983.58.6.0949

[299] Smith ER , Scott RM . Surgical management of moyamoya syndrome. Skull Base. 2005;15(1):15‐26. 16148981 10.1055/s-2005-868160 PMC1151701

[300] Miyamoto S , Yoshimoto T , Hashimoto N , et al. Effects of extracranial‐intracranial bypass for patients with hemorrhagic moyamoya disease: results of the Japan Adult Moyamoya Trial. Stroke. 2014;45(5):1415‐1421. 24668203 10.1161/STROKEAHA.113.004386

[301] Noshiro S , Mikami T , Komatsu K , et al. Neuromodulatory role of revascularization surgery in moyamoya disease. World Neurosurg. 2016;91:473‐482. 27150656 10.1016/j.wneu.2016.04.087

[302] Qian C , Yu X , Li J , Chen J , Wang L , Chen G . The efficacy of surgical treatment for the secondary prevention of stroke in symptomatic moyamoya disease: a meta‐analysis. Medicine (Baltimore). 2015;94(49):e2218. 26656359 10.1097/MD.0000000000002218 PMC5008504

